# Novel archaeal ribosome dimerization factor facilitating unique 30S–30S dimerization

**DOI:** 10.1093/nar/gkae1324

**Published:** 2025-01-11

**Authors:** Ahmed H Hassan, Matyas Pinkas, Chiaki Yaeshima, Sonoko Ishino, Toshio Uchiumi, Kosuke Ito, Gabriel Demo

**Affiliations:** Central European Institute of Technology, Masaryk University, Kamenice 5, Brno 625 00, Czech Republic; Central European Institute of Technology, Masaryk University, Kamenice 5, Brno 625 00, Czech Republic; Department of Biology, Faculty of Science, Niigata University, 8050 Ikarashi 2-no-cho, Niigata 950-2181, Japan; Department of Bioscience and Biotechnology, Faculty of Agriculture, Kyushu University, 744 Motooka Nishi-ku, Fukuoka 819-0395, Japan; Department of Biology, Faculty of Science, Niigata University, 8050 Ikarashi 2-no-cho, Niigata 950-2181, Japan; Department of Biology, Faculty of Science, Niigata University, 8050 Ikarashi 2-no-cho, Niigata 950-2181, Japan; Central European Institute of Technology, Masaryk University, Kamenice 5, Brno 625 00, Czech Republic

## Abstract

Protein synthesis (translation) consumes a substantial proportion of cellular resources, prompting specialized mechanisms to reduce translation under adverse conditions. Ribosome inactivation often involves ribosome-interacting proteins. In both bacteria and eukaryotes, various ribosome-interacting proteins facilitate ribosome dimerization or hibernation, and/or prevent ribosomal subunits from associating, enabling the organisms to adapt to stress. Despite extensive studies on bacteria and eukaryotes, understanding factor-mediated ribosome dimerization or anti-association in archaea remains elusive. Here, we present cryo-electron microscopy structures of an archaeal 30S dimer complexed with an archaeal ribosome dimerization factor (designated aRDF), from *Pyrococcus furiosus*, resolved at a resolution of 3.2 Å. The complex features two 30S subunits stabilized by aRDF homodimers in a unique head-to-body architecture, which differs from the disome architecture observed during hibernation in bacteria and eukaryotes. aRDF interacts directly with eS32 ribosomal protein, which is essential for subunit association. The binding mode of aRDF elucidates its anti-association properties, which prevent the assembly of archaeal 70S ribosomes.

## Graphical Abstract

**Graphical Abstract:**
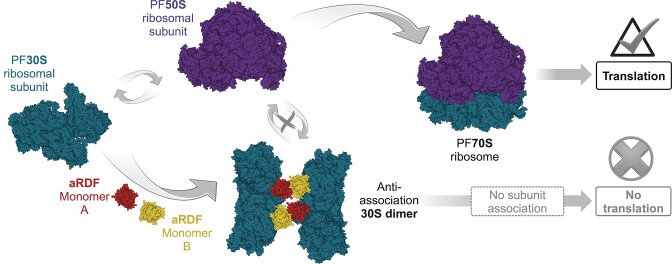

## Introduction

Ribosomes, the molecular machines responsible for protein synthesis, are central to the fundamental processes of life (1). However, their activity comes with a significant energy cost (2), necessitating mechanisms to regulate their function and conserve cellular resources under stress or adverse conditions (3,4).

One such regulatory strategy is ribosome hibernation (5), in which ribosomes enter a dormant state during environmental stress or growth phase transitions. In bacteria, ribosome hibernation mechanisms either inactivate individual 70S ribosomes (6–8) or promote the formation of 100S ribosomes—functionally inactive dimers of 70S ribosomes (9–16). In *Escherichia coli* and other gamma-proteobacteria, ribosome modulation factor (RMF) and hibernation promoting factor (HPF) form hibernating 100S ribosomes (9,15,16). RMF binds between the head and platform of the 30S small ribosomal subunit (9), while HPF extends across the A- and P-sites on the 30S subunit (9), blocking transfer RNA (tRNA) binding and other translation factors (7). The formation of hibernating 100S ribosomes is further mediated by the ribosomal protein uS2, which leads to the simultaneous deactivation of 70S ribosomes within the 100S hibernation complex (9–11,13,14). The involvement of uS2, along with the binding positions of the hibernation factors, blocks the messenger RNA (mRNA) pathway, preventing mRNA binding and rendering the 100S ribosomes translationally inactive. In contrast some bacteria, such as *Bacillus subtilis*, *Lactococcus lactis*, *Thermus thermophilus* and *Staphylococcus aureus*, use only a long variant of HPF (lHPF) (10–14), which forms a homodimer interface of two 30S subunits to trigger ribosome dimerization.

Eukaryotic hibernation mechanisms, while functionally similar to those in bacteria, are controlled by distinct, non-homologous proteins. In yeast, studies of hibernating 80S ribosomes have shown that the protein Lso2 binds to the P-site of the 40S subunit and the A- and P-sites of the 60S subunit (17), effectively blocking the binding of mRNA and tRNA and inducing 80S ribosome hibernation. Additionally, in *Saccharomyces cerevisiae*, the protein Stm1p plays a key role in 80S ribosome hibernation. Stm1p binds within the intersubunit space between the 40S and 60S subunits, with its N-terminal helix anchored in the mRNA exit channel of the 40S subunit while interacting with the central protuberance of the 60S subunit (18). This dual interaction stabilizes the ribosome in a dormant state, preventing subunit dissociation and mRNA binding. In mammals, hibernation factors homologous to their yeast counterparts have been identified, with their function depending on the conformational state of the 80S ribosome—whether it is in the non-rotated or rotated state. In the non-rotated state, the Lso2 homolog CCDC124 blocks the mRNA entry channel (17), whereas in the rotated state, the SERBP1 protein, a homolog of yeast Stm1p, obstructs the A- and P-sites on the 40S subunit (17). Moreover, EBP1 was found to bind to the peptide exit tunnel of hibernating human ribosomes independently of the ribosome’s rotational state or the presence of hibernation factors SERBP1 and CCDC124, thereby obstructing the binding site for co-translational factors and defining EBP1 as a hibernation factor (17). More recently, ribosomal hibernation in its dimeric form was structurally characterized in the microsporidian species *Spraguea lophii* (19). In this organism, ‘100S’ ribosome dimers form independently upon entry into host cells, without the involvement of hibernation factors. The dimer interface in *S. lophii* is located in the beak of the 40S subunit head, leaving the mRNA path and tRNA binding regions unobstructed.

In addition to ribosome hibernation, ribosome anti-association mechanisms also play a critical role in controlling translation by preventing premature or inappropriate assembly of the ribosomal subunits (3,4). In bacteria, the initiation factor 3 (IF3) serves as a central player in ribosome assembly (20,21). IF3 specifically targets the 30S ribosomal subunit, interacting primarily with its C-terminal domain in proximity to the helix 44 (h44) of the 16S rRNA (20,22). This interaction blocks the premature association of the 30S subunit with the 50S subunit (22). By preventing the ribosomal subunit association, IF3 ensures proper decoding of mRNA and modulates translation initiation rates (23–25), thereby playing a pivotal role in ribosome assembly and translation regulation.

In eukaryotes, the multi-subunit complex of eukaryotic IF3 (eIF3; absent in archaea) performs a similar anti-association function, but with additional regulatory roles (26). While eIF3’s primary function is to promote translation initiation by facilitating the recruitment of the 40S ribosomal subunit to the mRNA and assembling the 43S pre-initiation complex, it can also act as an anti-association factor (27). Under certain stress conditions, eIF3 binds to the 40S subunit and inhibits its association with the 60S subunit, thereby regulating the initiation of translation. This highlights the dual role of eIF3, both as a facilitator of translation under normal conditions and as a suppressor under stress, contributing to translational control in eukaryotic cells (26).

In contrast, archaeal translation regulation remains less well understood. While ribosome hibernation and anti-association mechanisms are well characterized in bacteria and eukaryotes, archaea have been largely understudied in this context. One recent discovery in the hyperthermophilic archaeon *Pyrococcus furiosus* (28) has shed light on a potential ribosome-associated factor: PF0560, a novel ribosome dimerization factor. PF0560 mediates the formation of 30S–30S dimers (28) and suppresses the formation of 70S ribosomes (28), which are essential for translation. This dimerization likely functions as an adaptation to extreme environmental conditions, under which *P. furiosus* thrives. However, the structural and mechanistic details of how PF0560 facilitates 30S–30S dimer formation remain largely unknown.

In this study, we present cryo-electron microscopy (cryo-EM) structures of dimerized small ribosomal subunits (30S) from *P. furiosus*, revealing a unique dimerization interface facilitated by a novel archaeal ribosome dimerization factor (aRDF; previously known as PF0560). aRDF forms two homodimers upon interacting with two 30S subunits, establishing a head-to-body dimer architecture of the two 30S subunits. Positioned close to the platform of 30S subunits near the neck region, the interaction surface of aRDF dimers predominantly covers the interaction with 16S rRNA of the 30S subunits. Additionally, aRDF dimers directly interact with the conserved eS32 ribosomal protein situated in the intersubunit surface region of the 30S subunits, crucial for intersubunit bridge formation to initiate ribosome subunit joining. The structural architecture of the 30S dimers, along with the protein’s ability to suppress the formation of *P. furiosus* 70S ribosomes, classifies the aRDF protein mainly as an archaeal ribosome anti-association factor that inhibits ribosome subunit joining.

## Materials and methods

### Archaeal ribosome purification

*Pyrococcus furiosus* cells were grown anaerobically at 98°C (28,29). The cells were ground with twice their weight of alumina and DNase (5 U/g cell pellet; Takara Bio Co.) at 4°C for 30 min, and then suspended in buffer A [20 mM Tris–HCl, pH 7.6, 10 mM MgCl_2_, 40 mM NH_4_Cl, 0.5 mM spermine and 2 mM dithiothreitol (DTT)]. The disrupted cell sample was centrifuged at 11 000 rpm for 30 min to get rid of cell debris and alumina. The cell extract was layered on a linear sucrose gradient (10–30%) containing buffer A. The gradients were centrifuged for 6.3 h at 27 500 rpm in a Hitachi P28S rotor, and the ribosome fraction of each gradient was pooled together and then pelleted at 45 000 rpm for 16 h using a Hitachi P50AT2 rotor. After removing the ribosome-associated proteins by incubating the ribosome fraction with buffer B (20 mM Tris–HCl, pH 7.6, 10 mM MgCl_2_, 1 M NH_4_Cl, 0.5 mM spermine and 2 mM DTT), the 70S ribosomes were isolated using a linear sucrose gradient (10–28%) in buffer A. The samples were centrifuged at 24 000 rpm at 4°C for 12 h in a Hitachi P28S rotor, following the protocol described previously (28). To isolate the 30S and 50S ribosomal subunits, a similar linear sucrose gradient centrifugation was performed in SB buffer (20 mM Tris–HCl, pH 7.6, 10 mM MgCl_2_, 0.5 M NH_4_Cl and 2 mM DTT), using the same rotor settings as for the 70S ribosomes. The ribosomal fractions were pooled and collected by 40 000 rpm centrifugation for 22 h at 4°C in a Hitachi P50AT2 rotor. The purified ribosomal samples were stored in buffer A containing 50% glycerol at −80°C prior to use.

### aRDF protein purification

The gene that encodes aRDF (PF0560 protein) was cloned into the pET21b vector (ampicillin resistance), purified with a C-terminal 6× His-tag and expressed in *E. coli* BL21(DE3) cells as described previously (28). The protein was expressed for 3 h at 37°C, after induction with 0.5 mM isopropyl β-d-1-thiogalactopyranoside at an OD_600_ of 0.5. The cells were harvested by centrifugation for 10 min at 5000 rpm. The cells were resuspended in buffer A [20 mM HEPES–KOH, pH 7.6, 1 M NH_4_Cl, 5% (v/v) glycerol and 7 mM β-mercaptoethanol (β-ME)] and disrupted using sonication. Centrifugation was done at 11 000 rpm to get rid of insoluble materials and cell debris. Afterward, the supernatant was heated at 70°C for 10 min, and then centrifuged at 33 000 rpm using a Hitachi P50AT2 rotor for 30 min to remove insoluble material. The supernatant was heated again for 20 min at 80°C to remove all *E. coli*-related components. The purification was done using both Ni-NTA affinity purification and heparin affinity purification. The sample was first loaded onto a cOmplete His-Tag Purification Resin (Roche) equilibrated with buffer A, washed with buffer A containing 10 mM imidazole and then eluted with buffer A containing 300 mM imidazole. Subsequently, the fractions containing aRDF were collected and dialyzed against buffer B (20 mM Tris–HCl, pH 7.6, 300 mM NaCl, 7 mM β-ME). The dialyzed sample was loaded into a HiTrap Heparin HP column (Cytiva) equilibrated with buffer B, washed with buffer B and then eluted with a linear gradient with buffer B containing 2 M NaCl. Finally, the purified sample was dialyzed against buffer C (20 mM Tris–HCl, pH 7.6, 300 mM NaCl, 7 mM β-ME), concentrated using an Amicon Ultra-15 centrifugal concentrator (10 kDa cutoff, Millipore) and stored at −80°C prior to use.

### Cross-linking aRDF and the 30S–aRDF dimer

The amine-reactive, homobifunctional, cross-linker disuccinimidyl diacetic urea (CF PLUS Chemicals) and disuccinimidyl dibutyric urea (CF PLUS Chemicals) were used in the cross-linking experiments. The reactions of the different cross-linkers were conducted in the same manner. Cross-linking reaction mixtures contained 10 μM 30S subunit (30S) and aRDF, or 5 μM aRDF alone in reaction buffer [20 mM HEPES–KOH, pH 7.0, 12.5 mM MgCl_2_, 100 mM NH_4_Cl and 0.5 mM ethylenediaminetetraacetic acid (EDTA)]. Using a 100-fold molar excess of the cross-linker over 30S and aRDF, a stock solution of the cross-linker (1 M) was freshly prepared in dimethyl sulfoxide after 10 min of incubation at room temperature to avoid moisture sensitivity problems. After 5 min of incubation at 70°C of 30S and aRDF, or aRDF alone, the cross-linking reaction was carried out at room temperature for 30 min. The reaction was stopped by the addition of 20 mM Tris–HCl, pH 8.0 (final concentration). The samples were loaded onto a 12% sodiumdodecyl sulfate–polyacrylamide gel electrophoresis, and the high-molecular-weight protein species, including aRDF dimer and the 30S–aRDF complex, were excised from the gel.

### LC–MS/MS and analysis

After destaining and washing procedures, the proteins in the excised bands were subjected to reduction with DTT, alkylation with iodoacetamide and then incubated with trypsin (sequencing grade; Promega) for 2 h at 40°C, and subsequently with Glu-C (Roche) for 16 h at 25°C. Peptides extracted from gel pieces were analyzed by liquid chromatography–tandem mass spectrometry (LC–MS/MS) performed using an UltiMate 3000 RSLCnano system (Thermo Fischer Scientific) on-line coupled with a timsTOF Pro 2 spectrometer (Bruker). MS/MS data were searched against a custom database of relevant protein sequences in combination with the common Repository of Adventitious Proteins (cRAP) contaminant database using an in-house Mascot search engine (Matrixscience; version 2.6). Carbamidomethylation (C), propionamidation (C) and oxidation (M) were set as variable modifications in all searches.

The analysis of the cross-linking products was performed using MeroX (version 2.0) (30). The settings for C-terminal cleavage for trypsin were set at Lys and Arg residues for trypsin, and for GluC at Asp and Glu residues. Additionally, three missed cleavage sites were allowed. The sites for cross-linking were defined as Lys for site 1, and Lys, Ser, Thr, Tyr and N-terminus for site 2. All the cross-links were manually inspected using PyMOL (The PyMOL Molecular Graphics System, version 2.3.1, Schrödinger, LLC).

### Cryo-EM grid preparation, data collection and image processing

30S ribosomal subunits were mixed with aRDF in a 1:5 molar ratio by adjusting the concentration to 1 μM 30S with 5 μM aRDF (final concentrations) in reaction buffer (20 mM HEPES–KOH, pH 7.0, 12 mM MgCl_2_, 100 mM NH_4_Cl and 0.5 mM EDTA) in a 20 μl total volume. Afterward, the samples were incubated for 15 min at 70°C before being applied onto the grid. Quantifoil R2/1 300 mesh grids were glow discharged for 40 s at 40 W power with a 5 W range (Gatan Solaris II). A 3.5 μl drop of the 30S–aRDF complex was applied directly onto the grid and flash-frozen in liquid ethane using an FEI Vitrobot Mark IV (Thermo Fischer Scientific). Grids were blotted with blotting power 5 at 4.5°C and ∼95% humidity. The same procedure was applied to the 30S control sample (PF30S control) and to the 30S (1 and 0.3 μM) and 70S (0.2 μM) samples, establishing a concentration gradient of aRDF, with ∼5-fold and ∼65-fold excess, to evaluate aRDF’s anti-association and dimerization potential. Screening images of the 30S samples with aRDF were acquired using a Talos Arctica microscope operating at 200 kV, equipped with a K2 direct electron detector (Gatan Inc.). The nominal magnification was set to 100 000, resulting in a pixel size of 0.783 Å. The total dose per screening image was 1.14 e^−^/Å^2^. For the 70S samples with aRDF, screening images were captured on a Glacios microscope, also operating at 200 kV with a Falcon IV direct electron detector (Thermo Fischer Scientific). In this case, a nominal magnification of 120 000 was used, corresponding to a pixel size of 1.143 Å and the total dose per screening image was 1.2 e^−^/Å^2^.

A dataset of 11 033 micrographs was collected for the 30S–aRDF complex (1:5 ratio) in a Titan Krios microscope operating at 300 kV, equipped with a K3 direct electron detector and a BioQuantum K3 imaging filter with a slit width of 10 eV (Gatan Inc.), within a defocus range of −0.8 to −1.8 μm. The data acquisition was performed using SerialEM (31). Each exposure was acquired with continuous frame streaming at 40 frames per movie, and a total dose of 40 e^−^/Å^2^ per movie over a 2 s exposure. The nominal magnification was 105 000, which corresponds to a pixel size of 0.834 Å. The movies were motion corrected, and the frame averages were computed by incorporating all frames within each movie using MotionCorr2 (32). cisTEM (versus 1.0-beta) (33) was used to determine defocus values for each frame average and particle picking. Ninety-five movies with large drift or ice contamination were excluded from further analysis after inspection of the power spectra computed by CTFFIND4 (34) within the cisTEM software package. Initially, 792 586 particles were picked and used for 2D classification within cisTEM. The particles were classified into 50 classes in 20 cycles, and the 2D classes representing the 30S–aRDF dimer were selected. The stack of 203 335 particles was used to generate the *ab initio* 3D map reconstruction in cisTEM (33), based on the principle of iterative refinement, starting from random angular parameters (35). The *ab initio* map was used to fit the dimer of 30S subunits (PDB 4V6U) (36) without aRDF in Chimera (37). The goal was to generate an aRDF-free template for 3D classification and refinement in FREALIGN (35), in order to confirm that aRDF is genuinely bound to the dimer, given the unknown structural arrangement of aRDF within the complex. The particle stack and parameter file were exported from cisTEM, and 4×, 2× and 1× binned (the box size of 576 pixels) stacks were generated using resample.exe in the FREALIGN distribution (FREALIGN v9.11) (35).

The particle alignment, 3D classification and final refinement were performed in FREALIGN (35). The 4× binned particle stack was initially aligned in C1 symmetry to the generated non-biased reference using five cycles of mode 3 alignment (global search) in the resolution range of 300–30 Å. Subsequently, the 4× binned stack was aligned against the reference from the previous step using five cycles in mode 1 (local refine) in the resolution range 300–18 Å. The 2× binned image stack was afterward aligned to the common reference using mode 1 by increasing the high-resolution limit in a stepwise manner up until 8 Å (18, 12, 10 and 8 Å—five cycles per each resolution step). 3D density reconstruction was obtained using the 60% of particles with the highest scores. The refined particle parameters were used for 3D classification of the 2× binned stack into six classes in 50 cycles (resolution range of 300–8 Å) in C1 symmetry to detect any dynamic states of the 30S–aRDF complex structure. This classification revealed five high-resolution classes and one low-resolution (junk) class. Of the five high-resolution classes, three resembled Structure I and two Structure II of the 30S–30S dimer mediated by two aRDF homodimers. The particles assigned to the two sets of high-resolution classes were extracted from the 2× binned stack (with >50% occupancy and >0 score) using merge_classes.exe (part of FREALIGN distribution), resulting in sub-stacks of 113 596 (Structure I) and 63 890 (Structure II) particles, respectively. Further 3D classification of both particle sub-stacks using either a spherical mask or a 3D mask placed on the head of 30S subunits or aRDF homodimers did not find any additional classes nor better overall resolution for Structure I or II. The final refinements of the 1× binned sub-stacks to 8 Å resolution using mode 1 (five cycles) and the 95% of particles with the highest scores resulted in 3.2 Å maps (Fourier shell correlation; FSC = 0.143) for Structures I and II, respectively. These maps were used for model building and structure refinements. Local-resolution filtering was applied to the resulting cryo-EM maps, using blocres and blocfilt from the package Bsoft (versus 1.9.1) (38). The sharpening of the resulting cryo-EM maps was performed with bfactor.exe (35) using a constant *B*-factor of −80 Å^2^ to interpret high-resolution details of Structures I and II. FSC curves were calculated by FREALIGN for even and odd particle half-sets.

A dataset of 6697 micrographs was collected for the 30S control with a Talos Arctica microscope operating at 200 kV equipped with a K2 direct electron detector and a Bioquantum image filter with the slit width set to 20 eV (Gatan Inc.), within the defocus range of −0.8 to −1.8 μm. The data acquisition was performed using SerialEM (31). Each exposure was acquired with continuous frame streaming at 40 frames per movie, and a total dose of 40 e^−^/Å^2^ per movie. The nominal magnification was 165 000, corresponding to a pixel size of 0.783 Å. The methodologies for motion correction, particle picking and 2D classification were conducted by following the same procedures as described previously for the 30S–aRDF complex. Five hundred eighty-six movies with large drift or ice contamination were excluded from further analysis. Initially, 274 032 particles were picked and used for 2D classification. The final stack of 159 700 particles and the parameter file were exported from cisTEM, and 4×, 2× and 1× binned (the box size of 512 pixels) stacks were generated using resample.exe.

The particle alignment, 3D classification and final refinement were performed in FREALIGN (35) by following similar procedures to those described previously for the 30S–aRDF complex. The 4× binned particle stack was initially aligned to the generated reference of free 30S subunit (PDB 4V6U) (36). The refined particle parameters were used for 3D classification of the 2× binned stack into five classes in 50 cycles (resolution range of 300–8 Å) in C1 symmetry. This classification revealed one high-resolution class and four low-resolution (junk) classes. The high-resolution class showed the free 30S subunit, exhibiting partial occupancy of the tRNA within the P-site of the 30S subunit. The particles assigned to the high-resolution class were extracted from the 2× binned stack (with >50% occupancy and >0 score) using merge_classes.exe (part of FREALIGN distribution), resulting in a sub-stack of 40 051 particles. Further 3D classification was conducted using a spherical mask positioned within the P-site of the 30S subunit to elucidate the occupancy of the tRNA. This classification found two classes of free 30S subunits: one with the P-site tRNA present and another without it. The class comprising the free 30S subunit lacking bound tRNA (25 356 particles) was subsequently used in refinement as a control map for comparison with the 30S–aRDF complex. The final refinement of the 1× binned sub-stack to a resolution of 8 Å using mode 1 (five cycles) and the 95% of particles with the highest scores resulted in a 3.4 Å map (FSC = 0.143) for the 30S control. The map was used for model building and structure refinements. Local-resolution filtering was applied to the resulting cryo-EM map, using blocres and blocfilt from the package Bsoft (versus 1.9.1) (38). The sharpening of the resulting cryo-EM map was performed with bfactor.exe (35) using a constant *B*-factor of −80 Å^2^. FSC curves were calculated by FREALIGN for even and odd particle half-sets.

### Model building and refinement

Cryo-EM structures of the 70S ribosome from *P. furiosus* (PDB 4V6U) (36) and 30S subunit from *P. abyssi* (PDB 7ZHG) (39) were used as starting models for structure refinement. Multiple ribosomal proteins were modeled using structural models generated by AlphaFold (40). The aRDF protein was modeled using d-I-TASSER (https://zhanggroup.org//D-I-TASSER/) by supplying the sequence of the protein.

The models were initially fitted into cryo-EM maps using rigid-body fitting in Chimera (37). The manual modeling was performed using Coot (41). The linkers within the aRDF protein, particularly those regions poorly defined in the cryo-EM maps (such as the loops), were omitted from the model.

All structures were refined using phenix.real_space_refine in Phenix (42). Secondary-structure restraints and base-pairing restraints for ribosomal RNA (rRNA) were applied during the refinement process. Correlation coefficients (model-to-map fit in Phenix) were closely monitored to avoid overfitting the models to the corresponding maps. The refined structural models closely align with the corresponding maps, as shown by high correlation coefficients. FSC values between the final models and maps, as well as cross-validation half-map FSCs, were computed using Phenix, showing strong agreement between the structural models and maps. The resulting models have favorable stereochemical parameters, including minimal deviation from ideal bond lengths and angles, and a low number of macromolecular backbone outliers, as detailed in Supplementary Table S1. Structure quality was assessed using MolProbity (43). Structure superpositions and figure generation were performed using ChimeraX (44) and PyMOL. The analysis of close contacts at the interaction surfaces within the aRDF homodimer and between aRDF and 16S rRNA was performed using the software package PISA (45), which is part of the CCP4 suite (46).

### Sequence and structural analysis of aRDF and eS32 proteins

A phylogenetic tree was generated using a subset of fully sequenced genomes from Eukaryota and Archaea. NCBI TaxIDs of each species were retrieved from the NCBI database (https://lifemap-ncbi.univ-lyon1.fr) (47) and then imported into PhyloT-v2 (http://phylot.biobyte.de). The phylogenetic tree was visualized using iTOL version 6.9 (48). aRDF and similar proteins were explored manually through the NCBI BLAST (49) database and UniProt (50). Additionally, comparisons of the protein folds of aRDF and eS32 were investigated using the final structures as inputs for Foldseek (51). Multiple sequence alignments of proteins were done using DeepMSA (52). The ∼4600 non-redundant eS32 homolog sequences obtained were depicted using WebLogo 3 (53).

## Results

### The structural arrangement of the 30S–30S dimer facilitated by aRDF protein

**Figure 1.**
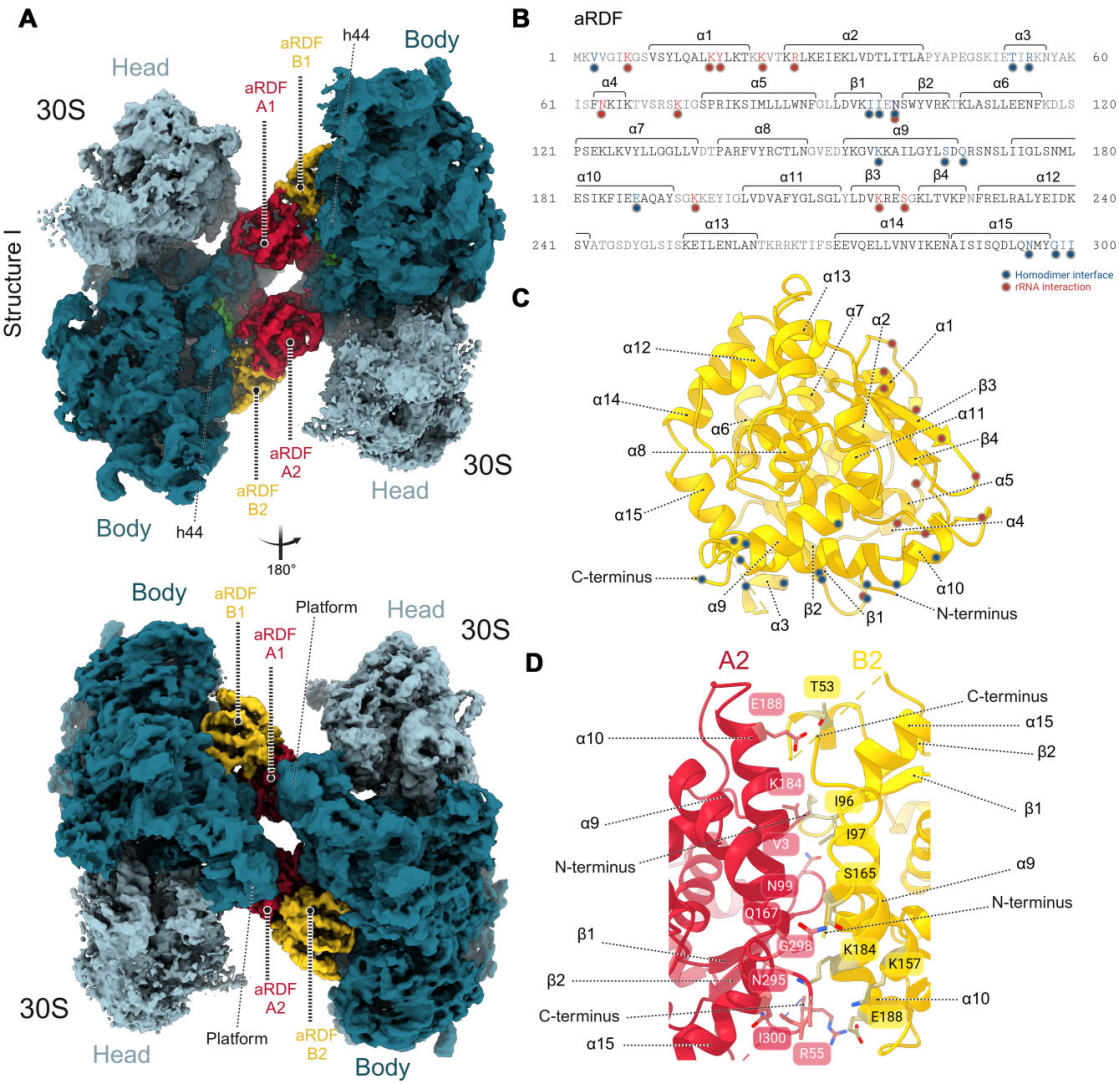
Cryo-EM structure of 30S–30S dimer formed by aRDF protein. (**A**) Original non-sharpened cryo-EM density map segmented into surface representation of the 30S–30S dimer structure with two aRDF homodimers. The map is displayed at 3.5*σ*. The 30S head is depicted in slate blue, the 30S body in teal and the aRDF monomers (A and B, of the respective homodimer) are shown in red (for monomer A) and gold (for monomer B). (**B**) aRDF protein sequence with secondary-structure elements indicated, including 15 α-helices and 4 β-strands. Residues involved in the aRDF homodimer interface are highlighted with a blue mark, while residues interacting with rRNA are highlighted with a red mark. (**C**) Structure of aRDF monomer with secondary structure indicated, showing interaction sites marked as described in panel (B). (**D**) Close-up view of the aRDF homodimer interface, showing selected residues in stick representation. The annotated residues form either polar or non-polar interactions between aRDF monomers A and B.

To explore the ability of aRDF to dimerize *P. furiosus* 30S subunits (28), we performed a structural analysis using cryo-EM by adding aRDF to a 30S subunit solution. The resulting cryo-EM maps (Structures I and II) (Supplementary Table S1 and Supplementary Figures S1 and S2) revealed a dimeric architecture comprising two small ribosomal subunits, mediated by two well-resolved homodimeric structures of the aRDF protein (Figure 1A and Supplementary Figure S3). A 3.2 Å resolution was sufficient to unambiguously assign the protein binding sites and the conformations of aRDF and 30S subunits (Supplementary Figures S2 and S3).

In this arrangement, the 30S–30S dimer is connected by two identical aRDF homodimers, each composed of monomers A and B, and remains unoccupied by mRNA, tRNAs or any translation factors. The model building of the aRDF protein, based on its known sequence (28), revealed its characteristic structure as an α-helical globular protein (Figure 1B and C, and Supplementary Figure S3). The interaction surface between the two monomers A and B of aRDF molecules mainly involves helices α9, α10 and α15, with their N-termini buried within this interface. The primary forces stabilizing this homodimeric interaction surface are non-polar interactions, supplemented by polar interactions originating from the side chains of residues predominantly located in helices α9, α10 and α15 (Figure 1B–D and Supplementary Table S2). The cross-linking experiments targeting Lys residues in the aRDF protein revealed detectable cross-links in the regions encompassing helices α3, α4, α5 and α10 (Supplementary Tables S3 and S4, and Supplementary Figures S4 and S5). These cross-links validated the accuracy of the aRDF model defined by the cryo-EM map of the aRDF-mediated 30S dimer and confirmed the orientation and interaction surface of the homodimeric aRDF architecture.

The surface of aRDF homodimeric molecules facing the 30S subunits is positively charged, which explains their predominant binding to 16S rRNA (Figure 1B, Supplementary Table S5 and Supplementary Figure S6). The monomers of aRDF homodimer interact differently with the 30S subunits (Figure 1A). Monomers A_1–2_ interact with both 30S subunits simultaneously binding at the platform regions. Monomers B_1–2_ interact solely with one 30S subunit binding between the platform and h44 of 16S rRNA. This unique interaction pattern generates a head-to-body architecture of the 30S–30S dimer, with 30S intersubunit interfaces facing each other. Interestingly, analysis of the cross-linking data for the 30S–aRDF complex revealed that cross-links only occurred within the aRDF molecules, suggesting that aRDF primarily recognizes and interacts with 16S rRNA segments within the 30S subunit, rather than engaging with ribosomal proteins (Supplementary Tables S3 and S4, and Supplementary Figures S4 and S5). To confirm the presence of all ribosomal proteins associated with the 30S subunit and ensure that the dimerized 30S subunits represent fully matured structures, a control sample consisting of the 30S subunit alone (PF30S control) was generated (Supplementary Table S1 and Supplementary Figures S2 and S7) and compared to the 30S–aRDF complex structure. This alignment confirmed the presence of all ribosomal proteins in the dimeric complex (Supplementary Figure S8A).

**Figure 2.**
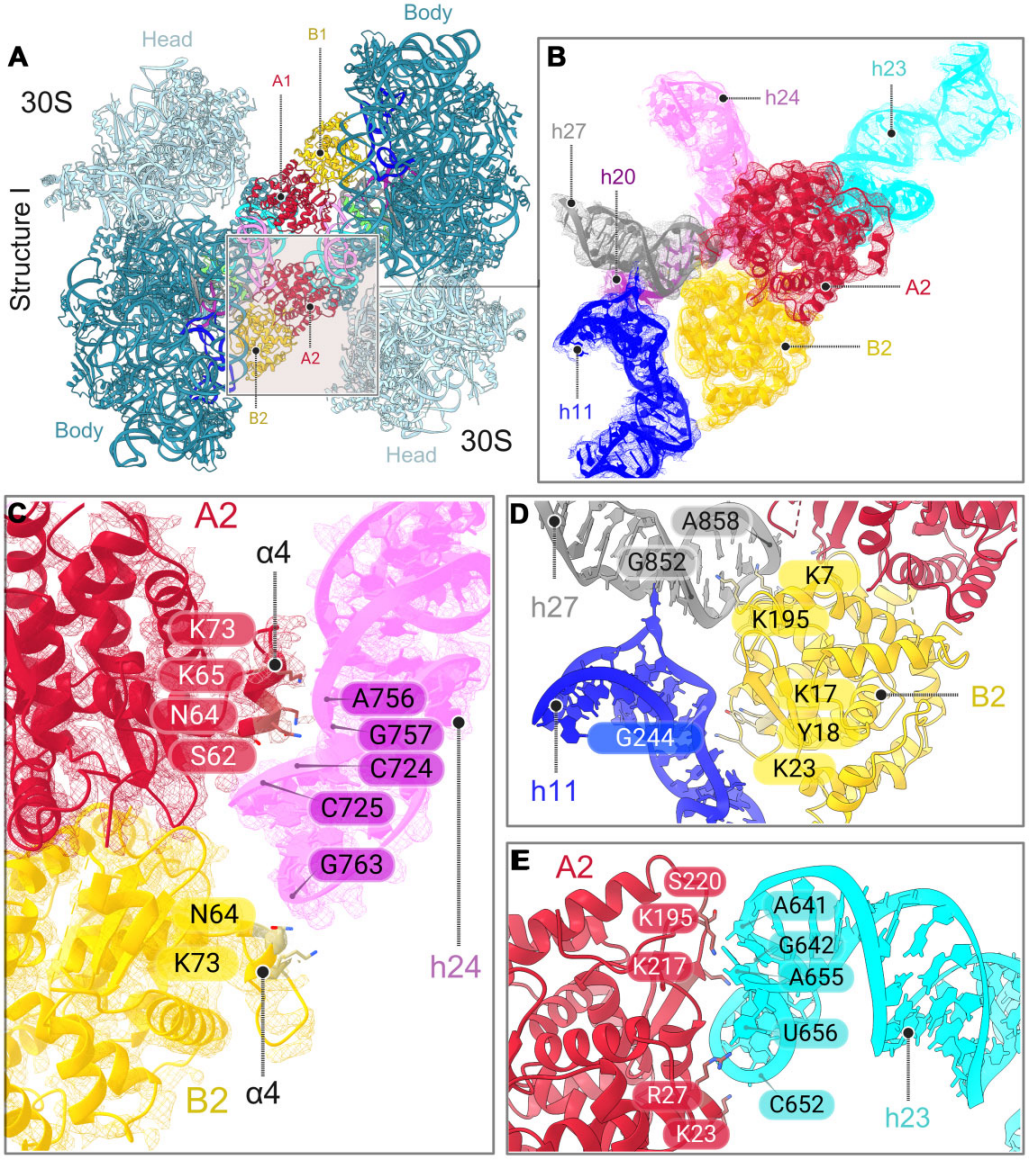
16S rRNA interaction with aRDF protein. (**A**) Structural representation of the 30S–30S complex with two aRDF homodimers. 16S rRNA helices in contact with the aRDF homodimer are colored as follows: h27 in gray, h20 in purple, h11 in dark blue, h24 in pink and h23 in cyan. eS32 ribosomal protein is in lime. (**B**) Structural view of 16S rRNA helices interacting with aRDF homodimer, with rRNA colored according to panel (A). The original non-sharpened cryo-EM map is displayed at 3.0*σ* and colored according to segments corresponding to the aRDF homodimer and rRNA helices. (**C**) Detailed structural view of aRDF homodimer forceps-like interaction with h24 of 16S rRNA. The original non-sharpened cryo-EM map is displayed at 3.5*σ* and colored according to segments corresponding to the aRDF homodimer and h24. (**D**) Detailed structural view of aRDF monomer B interaction with h11 and h27. (**E**) Detailed structural view of the interaction of aRDF monomer A with h23 of 16S rRNA. Only selected residues in the interface are shown. All aRDF residues are shown in stick representation, and the rRNA nucleotides are indicated.

As is mentioned earlier, both monomers A and B of aRDF engage with 16S rRNA in distinct ways (Figure 2A and B, Supplementary Table S5 and Supplementary Figure S6). Monomer A primarily interacts with h24 of one molecule of 30S subunit’s 16S rRNA, and h23 from the other molecule of 30S subunit’s 16S rRNA in the dimer (Figure 2B). Conversely, monomer B predominantly interacts with h11, h24 and h27 of the 16S rRNA from one 30S subunit (Figure 2B). The common binding region for the aRDF homodimer is h24 of 16S rRNA, where it binds in a forceps-like manner, providing a firm grip on the 16S rRNA (Figure 2C). This interaction is facilitated by α4 helices in both monomers, acting as anchors to grasp a portion of h24 in the 16S rRNA. Positively charged residues such as Lys or Arg, as well as other polar residues such as Asn or Ser, from monomers A and B of aRDF molecules, establish the interactions with h24 nucleotides (Figure 2C).

Additionally, within monomer B, a polar patch of residues situated in the α2 helix binds to h11 of the 16S rRNA (Figure 2D). Furthermore, Lys residues in monomer B, positioned in the loop regions connecting helical segments of the aRDF protein, interact with the backbone phosphates of nucleotides found in h27 of the 16S rRNA (Figure 2D). The interaction between aRDF monomer A and h23 involves several positively charged residues such as Lys and Arg, which interact with the phosphate backbone and bases of the 16S rRNA nucleotides (Figure 2E). Overall, comparing the 30S–30S dimer to the individual 30S subunit control sample, no local changes in the 16S rRNA were detected upon aRDF binding. This suggests that aRDF recognizes a mature 30S particle and does not alter the structure of the 30S subunit (Supplementary Figure S8A).

The analysis of the cryo-EM data using maximum-likelihood classification revealed two distinct conformations (Structures I and II) of the aRDF-mediated 30S–30S dimer (Supplementary Figures S1 and S2). The two structures differ by a slight movement of the 30S subunits toward the central axis of the dimeric architecture, using the two aRDF homodimers as anchoring points (Supplementary Figure S8C and D). Specifically, the 30S subunits were displaced by ∼3 Å toward the center of the dimeric structure, accompanied by a rotation of the 30S head domains by ∼4° toward the aRDF homodimers (Supplementary Figure S8D). The head domains of the 30S subunits exhibited a lower local resolution in both Structures I and II (Supplementary Figure S2), indicating a degree of flexibility. This finding is consistent with the observed rotations of the heads when comparing both structures, and it correlates with the binding regions of aRDF, which do not hinder the rotation of the 30S head domains. Such flexibility is anticipated, given the absence of mRNA or tRNAs bound to the 30S subunits in the dimeric structure. Although the relative positions of 30S subunits vary between Structures I and II, the 30S conformations within each dimer remain similar to each other.

### aRDF protein acts as an anti-association factor

The biochemical study revealed that aRDF is a ribosome-associated protein, but it does not form a protein-free 16S rRNA dimer, suggesting that it requires a unique structural architecture within the 30S subunit (28). This structure likely involves a specific region of the 16S rRNA and possibly ribosomal proteins that stabilize the aRDF homodimer on the 30S subunit.

**Figure 3.**
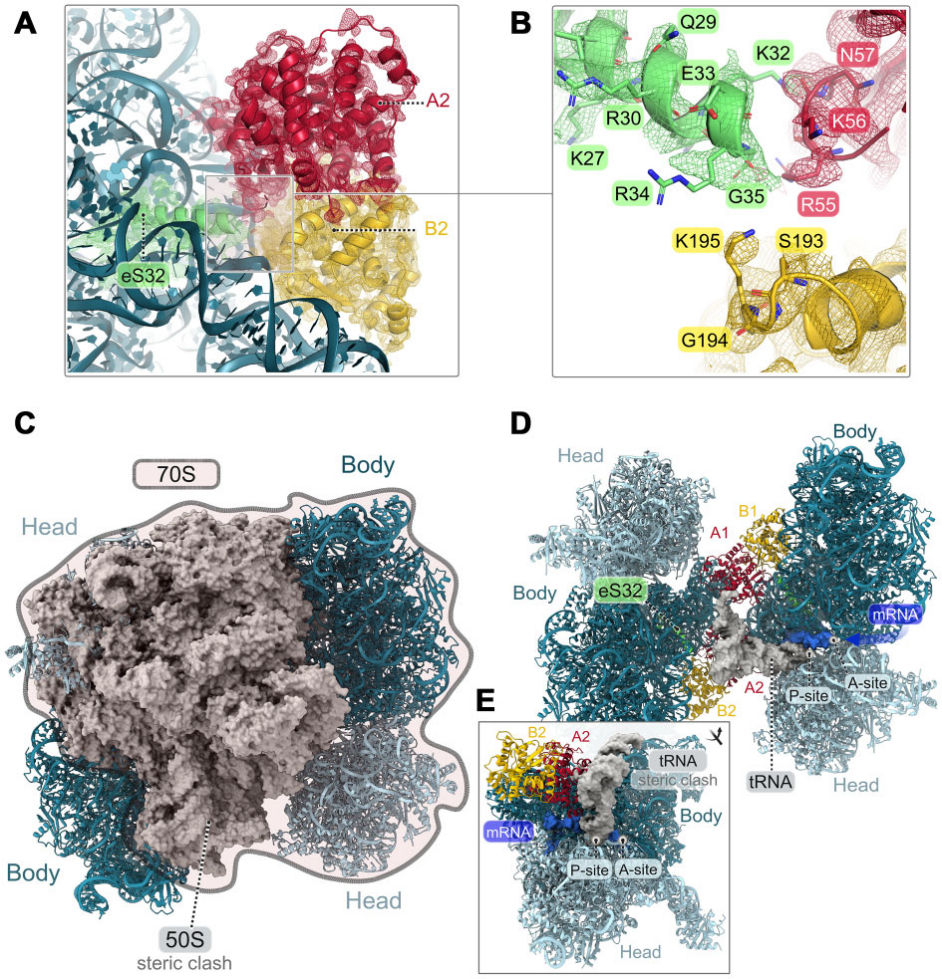
Binding mode of aRDF as an anti-association factor. (**A**) Interaction of aRDF’s homodimer with eS32 ribosomal protein. Monomer A of aRDF is depicted in red, monomer B in gold and eS32 in lime. The cryo-EM map was sharpened by applying a *B*-factor of −80 Å^2^ (displayed at 3.5*σ*) and colored according to segments corresponding to the aRDF homodimer and 30S. (**B**) Detailed view of the interface between eS32 and the aRDF homodimer, showing selected residues in the interaction interface represented as sticks. The flexible part of the α3 helix of monomer A2 is highlighted as a dashed line. The cryo-EM map was sharpened by applying a *B*-factor of −80 Å^2^ (displayed at 3.5*σ*) and colored according to panel (A). (**C**) Superimposition of the 30S–30S complex with aRDF on the 70S ribosome (the 50S subunit from PDB 4V6U is shown as a gray surface) (36). This reveals a significant steric clash between the 50S subunit and the second molecule of the 30S subunit from the dimeric structure. (**D**) Close-up view of the superimposition, aligned with the orientation in panel (C), displaying the positions of the P-site tRNA (as a gray surface) and mRNA (as a blue surface) derived from the superimposed 70S ribosome structure within the 30S–aRDF mediated complex. (**E**) Close-up, 30S head front view of the P-site tRNA and mRNA superimposition, highlighting the clash of the P-site tRNA with the homodimeric aRDF structure (A_2_B_2_) while showing an unobstructed path for mRNA in the presence of the aRDF homodimer.

Upon examination of the structure, the forceps-like binding mode of the aRDF homodimer on the 16S rRNA positions it near the C-terminus end of the eS32 ribosomal protein (Figure 3A and B). Although eS32 was initially associated with the large ribosomal subunit like eL41 protein (18), it has been shown to be structurally associated with the small ribosomal subunit and subsequently designated as eS32 ribosomal protein (54). To verify the involvement of the eS32 ribosomal protein in the 30S subunit, the control structure of the 30S subunit (PF30S control) consistently demonstrated the presence of eS32 associated with it (Supplementary Figure S8B). The proximity of eS32 to the decoding center implies its role in ensuring the fidelity of translation elongation (18). Within eukaryotic ribosomes, it is recognized that eS32 plays a crucial role in forming the intersubunit bridge, known as eB14, which resides at the center of the subunit interface (18,55). This bridge is essential for facilitating the association of ribosomal subunits, enabling the assembly of a fully functional ribosome (18).

The primary interaction interface between the C-terminus of eS32 and the aRDF homodimer is defined by the loop (Ser193–Lys195), which connects the α10 and α11 helices of aRDF’s monomer B and a segment of the α3 helix of aRDF’s monomer A (Figure 3B). The α3 helix (residues 52–57) exhibits significant disorder in monomer A compared to monomer B of the aRDF homodimeric structure, potentially facilitating coverage of the space required for intersubunit bridge formation. Indeed, the structural architecture of the 30S–30S dimer suggests that aRDF would hinder the association of the 50S subunit with the 30S subunit due to steric clashes (Figure 3C). Additionally, the structural architecture of the two aRDF homodimeric structures occupies the P- and E-site tRNA regions, likely inhibiting P-site tRNA binding (Figure 3D and E) but might allow mRNA accommodation, given the potential accessibility of the mRNA tunnel on the 30S subunit (Figure 3D and E). Despite the available structural data, cross-linking experiments did not find significant cross-links between eS32 and aRDF in the 30S–aRDF complex (Supplementary Tables S3 and S4, and Supplementary Figures S4 and S5). This is probably because eS32 is a short peptide that generates fragments that are too small and similar in mass to the background noise.

Moreover, our cryo-EM analysis provides direct visualization of how aRDF stabilizes 30S subunits, primarily by promoting 30S dimerization without inducing significant dissociation of 70S ribosomes (Supplementary Figure S9). When aRDF was added to isolated 30S subunits, we observed efficient 30S–30S dimerization, confirming its role in stabilizing these dimers (Supplementary Figure S9A and B). At substantially higher concentrations of aRDF, the 30S subunits aggregated more extensively, forming larger clusters (Supplementary Figure S9C and D). Furthermore, in samples containing 70S ribosomes, the addition of aRDF at a concentration ratio similar to that used for isolated 30S subunits resulted in minimal detection of isolated 30S and 50S subunits, while the levels of 70S ribosome particles remained similar to those in control samples without aRDF (Supplementary Figure S9E and F). Aggregation patterns resembling those observed with isolated 30S subunits were only seen at excessive aRDF concentrations (Supplementary Figure S9E and G). These findings align with previous biochemical data from sucrose sedimentation assays (28), which showed that adding aRDF to 30S subunits before the formation of 70S ribosomes reduced the 70S ribosome fraction and increased the 50S fraction, indicating a shift toward 30S–30S dimer formation (28). Additionally, in *P. furiosus* poly(U)-programmed translation assays, aRDF significantly inhibited polypeptide synthesis when added initially to the 30S subunit, reinforcing its role in preventing 30S and 50S subunit association (28). Together, these cryo-EM and biochemical data highlight aRDF’s function as an anti-association factor. Rather than promoting the dissociation of 70S ribosomes into subunits, aRDF facilitates 30S dimerization, suggesting that it plays a regulatory role in translation by modulating the assembly of ribosomal complexes.

### Homologs of aRDF and the conservation of eS32

**Figure 4.**
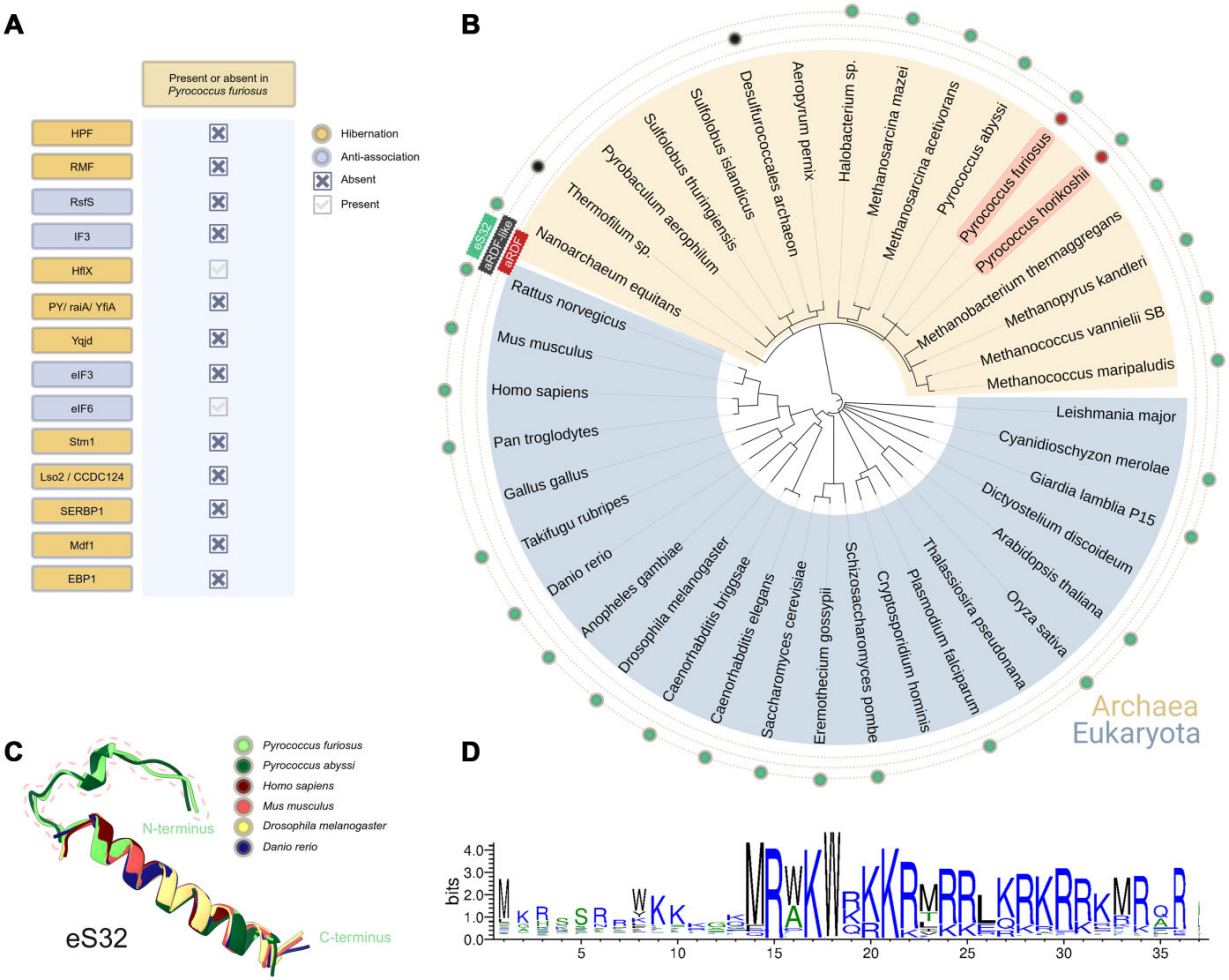
The presence of aRDF and eS32 in archaeal and eukaryotic species. (**A**) The availability of anti-association and hibernation factors in *P. furiosus*. Anti-association-related factors are depicted in light blue, while hibernation-related factors are shown in gold. Absent factors are marked with a cross, while present factors are marked with a check. (**B**) Phylogenetic tree illustrating representative eukaryotic (blue) and archaeal (orange) species. eS32 is highlighted in various species with a lime mark, aRDF with a red mark and aRDF-like uncharacterized proteins with a black mark. (**C**) Structural alignment of eS32 from different species. Dotted lines indicate the extension part of the N-terminus of *P. furiosus* and *P. abyssi* compared to other eukaryotic species. (**D**) Sequence alignment of eS32 from 4597 sequences. The alignment was performed using *P. furiosus* eS32 as the input sequence.

In our exploration of the genome of the archaeon *P. furiosus* for anti-association homologs of bacterial IF3 (25) or RsfS (25,56), and eukaryotic eIF3 (26) or eIF6 (57,58) factors, only the archaeal analogue of the 60S-binding protein eIF6 was detected, but no homologs of 30S- or 40S-binding proteins (Figure 4A). Examining the evolution of the aRDF protein in certain archaeal and eukaryotic species revealed that sequence-wise, this protein is exclusively present in the *Pyrococcus* subfamily (Figure 4B). Despite utilizing the structure of aRDF to search for structural similarities, no structurally available protein exhibited a comparable overall structure. We identified a few RNA- and DNA-binding proteins with similar folds in the N-terminus of aRDF, including LmrR from *L. lactis* (59), eS10 from *S. lophii* (19) and CodY from *B. subtilis* (60). Furthermore, we discovered five uncharacterized proteins from five distinct species with AlphaFold-predicted (40) structures highly resembling that of aRDF (Supplementary Figure S10). Notably, the top two matches originated from thermophilic archaea (*Desulfurococcales archaeon* and *Thermofilum* sp.) (Figure 4B), while the remaining three were from bacteria (*Acinetobacter piscicola*, *Clostridium butyricum* and *Clostridiales bacterium*). These findings suggest the potential existence of uncharacterized proteins in both archaea and bacteria that have a similar structure to aRDF protein.

When examining the dimerization capability of the aRDF protein in other species using bacterial 30S subunits from *E. coli* or eukaryotic 40S subunits from *Artemia salina* (28), none of them exhibited the dimerization observed with 30S subunits from *P. furiosus* (28). In both cases, the small ribosomal subunits lack homologs of the eS32 protein, suggesting that aRDF may require the presence of eS32 or a specific 16S rRNA fold/sequence to bind to. In certain bacterial and archaeal species, the eS32 homologs exhibit a similar sequence to that of *P. furiosus*. The elongated N-terminus of eS32 in *P. furiosus* is deeply embedded within the body of the 30S subunit (Figures 3A and 4C). It forms a hook-like amino acid stretch anchored between h27, h44 and h45 of the 16S rRNA, tightly binding it to the small ribosomal subunit. This structural characteristic was also observed in the archaeon *P. abyssi* (61) (Figure 4C) and the bacterium *Mycobacterium smegmatis* (bS22 ribosomal protein) (62). Sequence alignment of several thousand eS32 homologs revealed the absence of the extended N-terminus (Figure 4C and D), with a sequence comprising ∼25 amino acids. This sequence is highly conserved and positively charged (Figure 4D), facilitating tight interaction with rRNA. Although many commonly investigated archaeal and eukaryotic species contain eS32 ribosomal protein, none of them exhibited the presence of an aRDF homolog (Figure 4B). It is possible that these species employ a different ribosome-binding factor that functions similarly to the aRDF protein. This is supported by the fact that the main interaction surface of the aRDF homodimer is still defined by the C-terminus of the eS32 protein, which is highly conserved across numerous archaeal and eukaryotic species (Figure 4D).

## Discussion

**Figure 5.**
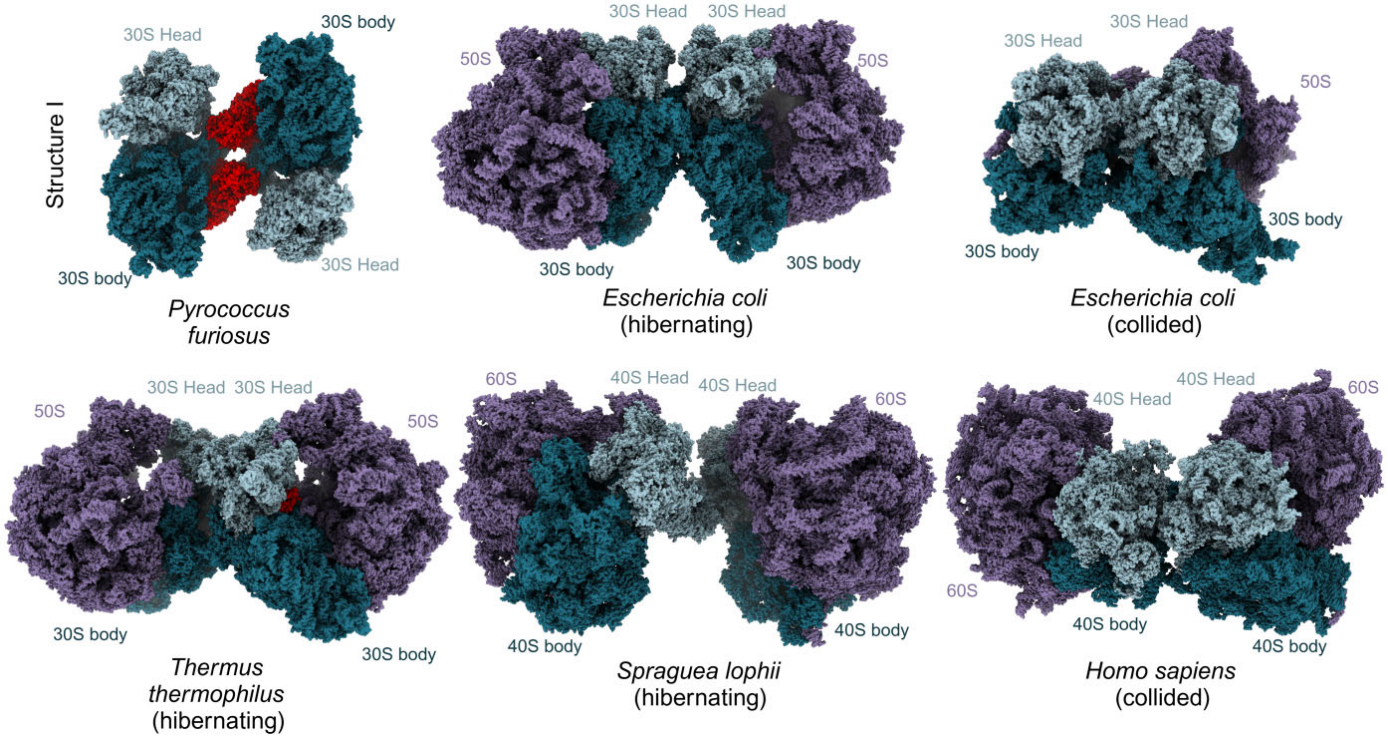
Distinctive architecture of archaeal ribosomal dimers compared to other disome structures. Surface structural representation of the 30S–30S dimer alongside hibernating and collided disomes from various species, depicted side by side. The factors responsible for dimerization are highlighted in red in all structures when visible in the specific orientation. The large subunit is shown in purple, the head of the small subunit in slate blue and the body of the small subunit in teal. The structures depicted are *E. coli* hibernating 100S (PDB 6H58) (9), *E. coli* collided disomes (PDB 8R3V) (66), *T. thermophilus* hibernating 100S (PDB 6GZX) (10), *S. lophii* hibernating dimer (PDB 8P60) (19) and *H. sapiens* collided disomes (PDB 7QVP) (65).

Our cryo-EM analysis offers new insights into the structural mechanism by which the aRDF in *P. furiosus* regulates translation through the formation of 30S–30S dimers. In both bacterial and eukaryotic systems, ribosome dimerization typically occurs during hibernation, involving a head-to-head arrangement of small ribosomal subunits (5,63). This configuration contrasts sharply with the head-to-body formation observed in this study (Figure 5). For instance, in bacteria, the 30S subunits adopt various head-to-head orientations within 100S dimers (9–11,13), stabilized by the interaction of ribosomal protein uS2, which interacts with the mRNA entry channel. The eukaryotic hibernation dimer in *S. lophii* forms a head-to-head interface involving ribosomal proteins eS31 and eS12 and the 16S rRNA, creating a connecting bridge at the small subunit’s beak (19). Aside from hibernation, ribosome dimerization can also occur as a response to translational stalling, forming disomes through ribosome collisions (64–66). Despite differing from hibernation-driven dimers, ribosome collisions also share the characteristic head-to-head interaction of 30S or 40S subunits (Figure 5). In bacterial disomes, this configuration is stabilized by interactions involving ribosomal proteins such as uS9, uS10 and uS2 (66). In higher eukaryotes, disome formation involves RACK1 of the leading ribosome and interactions between uS3, uS10 and eS10 of the colliding ribosome (64,65). Importantly, our findings reveal that no ribosomal proteins are involved in the direct interaction between the 30S subunits in the observed 30S–30S dimer, underscoring the distinctive nature of the aRDF-mediated interface.

Additionally, the ribosomal inactivation via hibernation in *E. coli* is mediated by RMF, which binds to the head and platform of the 30S subunit, blocking the A-site (9). The binding of HPF further reinforces the conformational changes induced by RMF, thereby obstructing mRNA and tRNA binding at both the A- and P-sites (9). Similarly, in the eukaryotic hibernation dimer of *S. lophii*, a density resembling the MDF1 protein blocks the E-site (19,67), holding the L1 stalk in a conformation that prevents tRNA binding. These features facilitate translation inhibition by preventing mRNA and tRNA access to critical ribosomal sites. In contrast, the head-to-body configuration in the 30S–aRDF complex features aRDF homodimers that facilitate dimer formation by blocking the E- and P-site tRNA binding sites on the 30S subunits while keeping the mRNA binding site accessible (Figure 3D and E). Notably, *P. furiosus* lacks homologs to bacterial or eukaryotic hibernation factors, such as RMF, HPF or MDF1 (Figure 4A). This absence suggests that the formation of the 30S–30S dimer is unlikely to be associated with canonical hibernation mechanisms. Instead, the distinctive head-to-body architecture facilitated by aRDF could provide an alternative strategy for translation regulation, specifically targeting small ribosomal subunits. This unique mode of dimerization highlights the adaptive versatility of ribosomal regulation mechanisms across different evolutionary branches and raises intriguing questions about the evolutionary pressures that drive such structural innovations.

**Figure 6.**
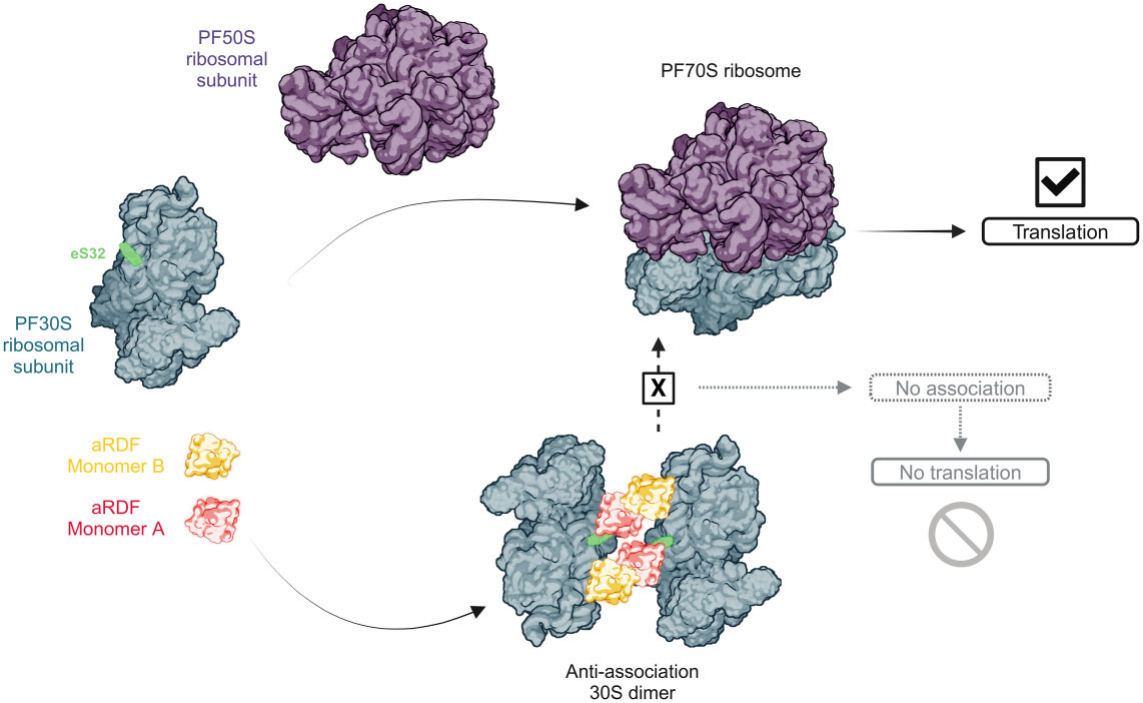
Scheme illustrating the potential role of aRDF as an anti-association factor regulating ribosome assembly and translation in *P. furiosus*.

A distinctive structural feature of aRDF is its specific interaction with the ribosomal protein eS32. The aRDF homodimer binds to this region in a forceps-like configuration that sterically obstructs the 50S subunit from associating with the 30S subunit by blocking critical intersubunit bridge region eB14 (18,55), effectively preventing the formation of functional 70S ribosomes. This interaction is consistent with biochemical evidence (28) identifying aRDF as an anti-association factor (Figure 6) that modulates translation by inhibiting ribosomal subunit assembly (28). The conserved nature of eS32 across archaeal and eukaryotic species (Figure 4B–D) suggests an evolutionarily shared mechanism of ribosomal regulation via anti-association function. Interestingly, while *P. furiosus* lacks other known homologous 30S- or 40S-binding anti-association factors (Figure 4A), aRDF appears to fulfill a similar role to bacterial IF3 (22,25) and eukaryotic eIF3 (25–27), both of which prevent premature ribosomal assembly by stabilizing the small subunit. In bacterial systems, IF3 binds the platform region of the 30S subunit, blocking intersubunit bridges to hinder ribosome formation (22). Specifically, IF3 targets the central pseudoknot formed by 16S rRNA helices h16 and h17 and can also interact with the decoding center, thus affecting mRNA and tRNA binding during initiation (22). In contrast, aRDF binds different regions of the 16S rRNA (helices h24, h23, h11 and h27) and forms a homodimer that stabilizes each 30S subunit by positioning them in a head-to-body orientation, effectively inhibiting access to the 50S subunit. Although the binding mode of aRDF differs from that of IF3, both factors ultimately prevent subunit association by targeting essential regions of the 30S subunit.

The anti-association role of aRDF also exhibits similarities to that of eIF3. Unlike aRDF, however, eIF3 also facilitates the recruitment of the 40S subunit to mRNA, promoting translation initiation (27,68). In contrast, aRDF’s function in *P. furiosus* appears to be limited to stabilizing the 30S subunit in an inactive state without engaging in mRNA recruitment, suggesting a more streamlined, archaeal-specific mechanism. While eIF3 operates as a multi-subunit complex (27,68), aRDF functions as a homodimer, demonstrating a simpler yet effective strategy for ribosome regulation in archaea, which may lack the complex regulatory machinery present in eukaryotes. Thus, aRDF may serve as an archaeal adaptation that mirrors the regulatory roles of IF3 and eIF3, preventing premature ribosomal assembly through a unique binding mode that stabilizes the 30S subunit, promoting efficient resource management under the specific environmental conditions of *P. furiosus*.

In addition to aRDF’s established role as an anti-association factor in *P. furiosus*, aRDF may play a role in ribosome recycling. Unlike bacteria, which rely on ribosome recycling factor and elongation factor G for ribosome recycling (69), archaea use a more streamlined system involving the ATP-binding cassette protein ABCE1 to mediate ribosome disassembly after translation termination (70). In *P. furiosus*, aRDF could complement this process by stabilizing ribosomal subunits, a strategy that aligns well with the simplified, efficient systems favored by extremophiles (71). In archaeal systems, ABCE1 collaborates with factors such as aPelota and aEF1α (72,73), which resemble eukaryotic ribosome recycling pathways. These factors interact with ABCE1 to recycle ribosomal subunits, allowing ribosomes to quickly return to an active state. The unique ability of aRDF to form 30S–30S dimers may be beneficial for conserving energy by maintaining ribosomal components in a state ready for reactivation. This structural stability offered by aRDF can provide protection against ribosomal degradation or misassembly, which is critical for survival in fluctuating extreme environments, such as those experienced by hyperthermophiles like *P. furiosus*.

Although proteomic studies found no significant upregulation of aRDF in response to heat or cold shock (66,74), it is plausible that aRDF is constitutively expressed, maintaining its role in modulating ribosome activity continuously without the need for stress-induced transcriptional responses. The presence of uncharacterized proteins in *Desulfurococcales* and *Thermofilum* species (Supplementary Figure S10), which closely resemble the structure of the aRDF protein, suggests an evolutionary trend toward streamlined yet efficient systems for ribosomal stability and preservation under extreme conditions.

In conclusion, the identification of the aRDF protein in *P. furiosus* underscores the adaptive versatility of ribosomal regulatory mechanisms, which differ from the hibernation and anti-association strategies seen in bacteria and eukaryotes. aRDF provides a streamlined and effective means of stabilizing ribosomes in archaea through the formation of 30S subunit homodimers. This stabilization of 30S–30S dimers exemplifies how archaeal ribosomes can achieve regulatory efficiency with minimal complexity. As such, aRDF is a potent ribosomal regulator, finely attuned to the environmental demands of *P. furiosus*. Future research may uncover similar regulatory factors in other archaeal species, which could further illuminate the distinct and domain-specific strategies employed for translation regulation. Such investigations could also have broader implications for our understanding of ribosomal function and regulation across various environmental contexts, contributing to the knowledge of how different organisms adapt to their unique habitats.

## Supplementary Material

gkae1324_Supplemental_File

## Data Availability

The EM density maps generated in this study have been deposited in the EMDB under accession codes EMD-50611 (Structure I), EMD-50612 (Structure II) and EMD-50613 (30S control). The atomic coordinates generated in this study have been deposited in the PDB under the accession codes 9FNY (Structure I), 9FNZ (Structure II) and 9FO0 (30S control).
