# Supplementary material for: Novel archaeal ribosome dimerization factor facilitating unique 30S–30S dimerization
Source: Nucleic Acids Res. 2025 Jan 11;53(2):gkae1324. doi: 10.1093/nar/gkae1324 (PMC11724365; doi:10.1093/nar/gkae1324)
Supplement: gkae1324_Supplemental_File [file gkae1324_supplemental_file.pdf]

Supplementary material

Novel archaeal ribosome dimerization factor facilitating unique 30S-30S dimerization

Ahmed H. Hassan[1], Matyas Pinkas[1], Chiaki Yaeshima[2], Sonoko Ishino[3], Toshio Uchiumi[2], Kosuke Ito[2], Gabriel Demo[1*]

Supplementary Table S1
Refinement statistics for cryo-EM Structure I, Structure II and PF30S control

	Structure I	Structure II	PF30S control
**PDBID**	9FNY	9FNZ	9FO0
**EMDB**	EMD-50611	EMD-50612	EMD-50613
**Data collection and processing**			
Magnification	105,000x	105,000x	165,000x
Voltage (kV)	300	300	200
Electron exposure (e–/Å^2)	40	40	40
Defocus range (μm)	-0.8-1.8	-0.8-1.8	-1.0-2.6
Pixel size (Å)	0.834	0.834	0.783
Symmetry imposed	C1	C1	C1
Initial particle (no.)	203,335	203,335	159,700
Final particle (no.)	113,596	63,890	25,356
Map resolution (Å)**	3.2	3.2	3.4
FSC threshold	0.143	0.143	0.143
**Refinement**			
Initial model used (PDB code)	4V6U	4V6U	4V6U
Model resolution (Å)	6.6	6.6	6.6
Correlation Coefficient (cc_mask)*	0.79	0.76	0.83
Map sharpening *B* factor (Å^2)	-80	-80	-80
Model composition*			
Non-hydrogen atoms	130,968	130,980	60,801
Protein residues	8,301	8,303	3,563
RNA residues	2,990	2,990	1,495
B factors (Å^2)*			
Protein	235.79	240.46	217.59
RNA	231.12	238.05	183.05
R.m.s. deviations*,§			
Bond lengths (Å)	0.007	0.006	0.004
Bond angles (°)	0.84	0.84	0.67
Validation#			
MolProbity score	1.75	1.80	1.61
Clashscore	7.43	8.57	6.48
Poor rotamers (%)	0.3	0.3	0.1
Ramachandran plot#			
Favored (%)	95.03	95.17	96.26
Allowed (%)	4.92	4.77	3.71
Disallowed (%)	0.05	0.06	0.03
Validation (RNA) #			
Good sugar pucker (%)	97.0	96.9	96.4
Good backbone (%)	80.4	78.5	80.9

* from Phenix
# from Molprobity
§ root mean square deviations

Supplementary Table S2
aRDF homodimer interface contacts. The interaction interface between monomer A and B in aRDF is annotated according to the amino acid (column 1 and 4), the number of residue on aRDF sequence (column 2 and 5), atom (column 3 and 6) and distance of contact in Å (column 7).

**Monomer A**			**Monomer B**			**Distance (Å)**
Residue	Number	Atom	Residue	Number	Atom	
GLU	188	OE2	THR	53	OG1	3.62
ASN	295	O	LYS	184	NZ	3.08
GLY	298	O	LYS	184	NZ	2.54
ILE	300	O	LYS	157	NZ	2.56
VAL	3	N	ILE	96	O	2.41
ARG	55	NH1	GLU	188	OE1	2.52
ASN	99	ND2	ILE	97	O	3.89
GLN	167	NE2	SER	165	O	2.58
ILE	300	O	LYS	157	NZ	2.56
ARG	55	NH1	GLU	188	OE2	3.70
ARG	55	NH1	GLU	188	OE1	2.52

Supplementary Table S3
DSAU cross-linking results. Cross-linked residues are numbered according to full polypeptide sequences of aRDF (column 1 and 3), sequences of cross-linked peptides (target cross-linked residues in bold) (column 2 and 4), approximate distance measured within one aRDF monomer in Å (column 5), approximate distance measured within one homodimer in Å (column 6), and approximate distance measured between two different homodimers in Å (column 7). Cross-links involving the aRDF monomer and a single homodimer originated from either the aRDF alone or the 30S-aRDF cross-linked bands. All cross-links between the two homodimers were present in the 30S-aRDF complex band.

**Number of residue 1**	**Peptide 1**	**Number of residue 2**	**Peptide 2**	**Distance within aRDF monomer Cα-Cα (Å)**	**Distance within aRDF homodimer Cα-Cα (Å)**	**Distance between two aRDF homodimers Cα-Cα (Å)**
76	SKIG**S**PR	60	NYA**K**ISF	14.1 – 14.6 Å	21.8 – 22.6 Å	impossible
65	ISFN**K**IK	76	SKIG**S**PR	10.6 – 10.8 Å	26.3 – 28.2 Å	impossible
73	S**K**IGSPR	70	IKTV**S**R	7.8 – 8.3 Å	35.4 – 39.1 Å	37.0 – 37.0 Å
29	L**K**EIEK	25	KV**T**KR	6.4 – 6.5 Å	impossible	21.3 – 21.7 Å
72	**S**KIGSPR	67	**IK**TVSR	6.0 – 7.3 Å	impossible	33.7 – 34.8 Å
76	SKIG**S**PR	29	L**K**EIEK	17.2 – 17.7 Å	impossible	34.9 – 35.0 Å
73	S**K**IGSPR	68	IK**T**VSR	6.7 – 7.1 Å	impossible	36.1 – 36.6 Å
76	SKIG**S**PR	26	KVT**K**R	17.5 – 17.8 Å	impossible	32.3 – 32.5 Å
65	ISFN**K**IK	29	L**K**EIEK	20.4 – 20.8 Å	impossible	33.4 – 33.6 Å

Supplementary Table S4

DSBU cross-linking results. Cross-linked residues are numbered according to full polypeptide sequences of aRDF (column 1 and 3), sequences of cross-linked peptides (target cross-linked residues in bold) (column 2 and 4), approximate distance measured within one aRDF monomer in Å (column 5), approximate distance measured within one homodimer in Å (column 6) and approximate distance measured between two different homodimers in Å (column 7). Cross-links involving the aRDF monomer and a single homodimer originated from either the aRDF alone or the 30S-aRDF cross-linked bands. All cross-links between the two homodimers were present in the 30S-aRDF complex band.

**Number of residue 1**	**Peptide 1**	**Number of residue 2**	**Peptide 2**	**Distance within aRDF monomer Cα-Cα (Å)**	**Distance within aRDF homodimer Cα-Cα (Å)**	**Distance between two aRDF homodimers Cα-Cα (Å)**
73	S**K**IGSPR	70	IKTV**S**R	7.8 – 8.5 Å	impossible	33.3 – 34.0 Å
29	L**K**EIEK	29	L**K**EIEK	impossible	impossible	21.5 Å
53	GSKIE**T**IR	195	AQAYSG**K**K	impossible	14.7 – 14.8 Å	impossible
240	ID**K**SVATGS<br>SDYGLSIS	264	NLANT**K**R	18.2 – 19.0 Å	impossible	impossible
73	S**K**IGSPR	68	IK**T**VSR	7.8 – 8.0 Å	impossible	impossible
253	IDKSVATGS<br>SDYGLSI**S**	264	NLANT**K**R	19.2 – 20.9 Å	impossible	impossible
53	GSKIE**T**IR	56	**K**NYAK	5.2 – 5.3 Å	≈ 40 Å	impossible

Supplementary Table S5

16S rRNA interaction with aRDF. The interaction interface between 16S rRNA and aRDF is annotated according to the nucleotide or amino acid (column 1 and 4), the number of nucleotide in the 16S rRNA or residue in aRDF sequence (column 2 and 5), atom (column 3 and 6) and distance of contact in Å (column 7).

**rRNA**			**aRDF**			**Distance (Å)**
Base	Number	Atom	Residue	Number	Atom	
A	858	OP1	LYS	7	NZ	2.40
G	244	OP2	LYS	17	NZ	3.72
G	244	OP2	TYR	18	OH	2.86
G	244	O6	LYS	23	NZ	2.75
U	245	O4	LYS	23	NZ	3.74
G	763	OP1	ASN	64	ND2	2.79
G	718	OP1	LYS	73	NZ	3.56
G	718	OP2	LYS	73	NZ	3.59
C	725	O2'	ASN	99	ND2	3.69
G	852	OP1	LYS	195	NZ	2.70
U	651	O3'	LYS	23	NZ	2.86
U	651	O2'	LYS	23	NZ	3.83
U	656	O4	ARG	27	NH2	2.27
U	656	OP2	LYS	195	NZ	3.58
G	642	O6	LYS	217	NZ	2.99
A	641	O2'	SER	220	N	3.33
A	641	OP2	SER	220	OG	3.53

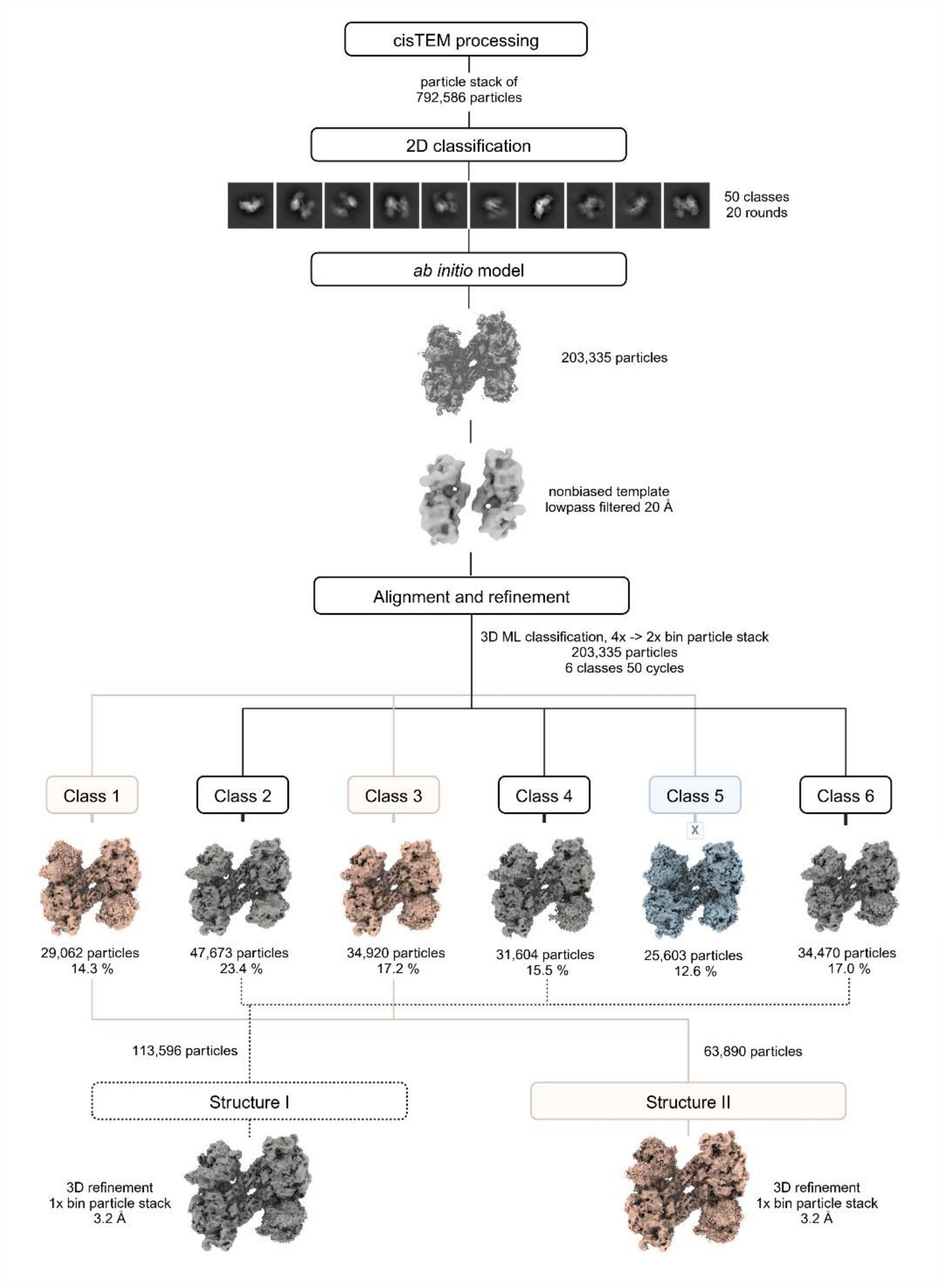

Supplementary Figure S1. Cryo-EM data classification scheme for 30S-30S dimer complexed with two aRDF homodimers. The final maps employed in structural modeling are depicted in grey (representing Structure I) and orange (representing Structure II), while the junk class 5 is indicated in blue.

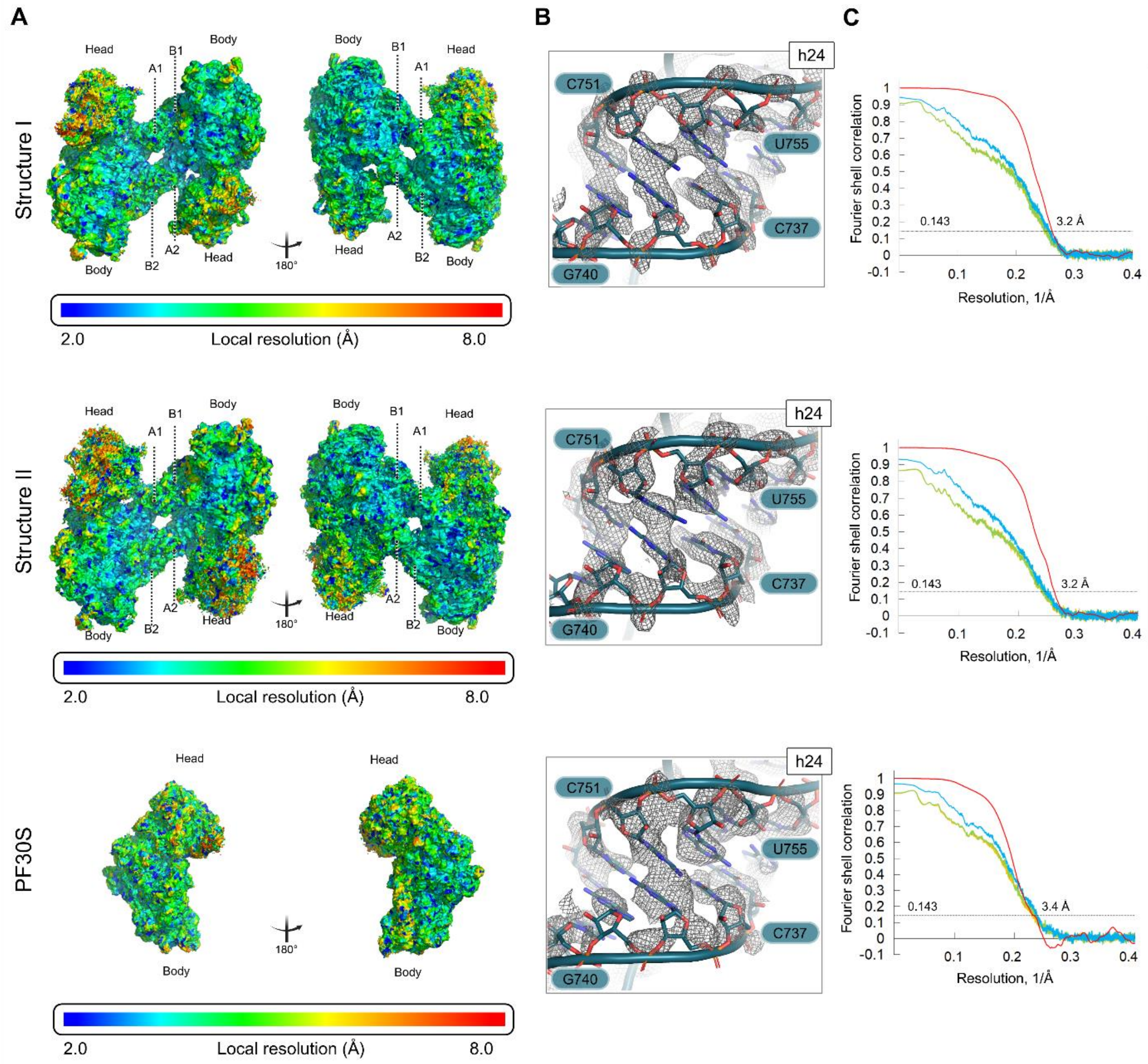

Supplementary Figure S2. Global and local resolution for Structure I, Structure II and PF30S control. (**A**) Local resolutions in cryo-EM maps for Structure I, Structure II and 30S control (PF30S). (**B**) Example of local map resolution for Structure I, Structure II and 30S control in the region of the h24 of 16S rRNA, located near the platform of the 30S subunit. The selected nucleotides of h24 of 16S rRNA are shown in stick representation. The maps (gray mesh) were sharpened by applying a B-factor of -80 Å^2 and are shown at 6.0 σ or 4.0 σ (for PF30S control). (**C**) Fourier shell correlation (FSC) between even- and odd-particle half maps (red) show that map resolutions range from 3.2 to 3.4 Å for Structure I, Structure II and 30S control (at FSC = 0.143, dotted line); FSC between final models and final maps (cyan), and cross-validation half-maps (half-map 1 in green and half-map 2 in orange) masked FSCs are also shown.

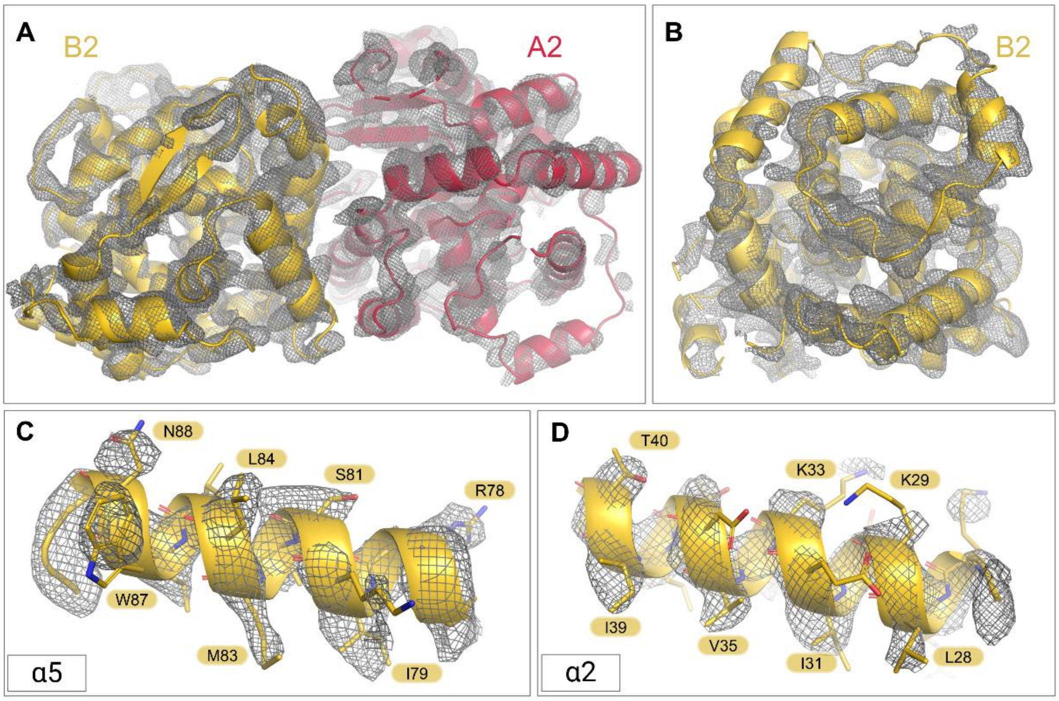

Supplementary Figure S3. Structure I - aRDF revealed by cryo-EM density map. (**A**) Depiction of the structural architecture of the aRDF homodimer (monomer A2 in red and monomer B2 in gold), fully defined by the cryo-EM density map. (**B**) Representation of aRDF monomer B2 in similar orientation as in Fig. 1C, enveloped by the cryo-EM density map. Detailed structural views of aRDF monomer B2, emphasizing specific residues in stick representation for α-helix 5 (**C**) and α-helix 2 (**D**) and their resolution within the cryo-EM density map. The maps (gray mesh) were sharpened by applying a B-factor of -80 Å^2 and are displayed at 3.5 σ.

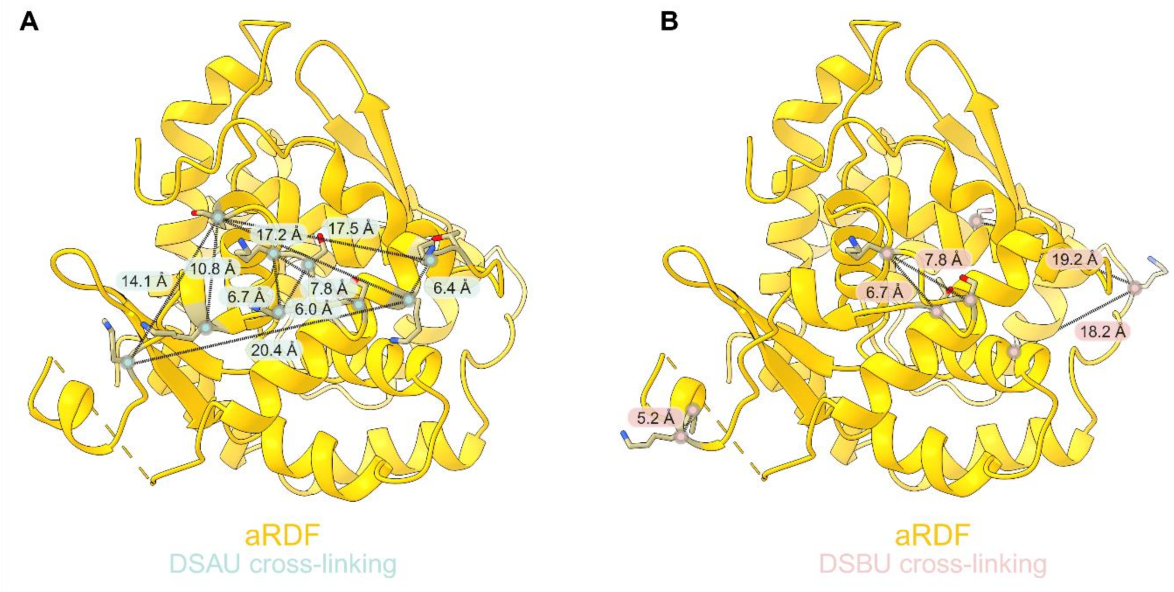

Supplementary Figure S4. Intraprotein cross-linking within a single molecule of aRDF. (**A**) DSAU cross-linking of aRDF molecule (cyan) and (**B**) DSBU cross-linking of aRDF molecule (pink). Selected cross-links are mapped onto the structure of aRDF, that are within the expected distance range of a cross-linker, with dotted lines indicating connections between residues and approximate distances in Å. These cross-linking experiments were conducted both for aRDF alone and aRDF within the 30S-aRDF complex; see also Supplementary Tables S3 and S4.

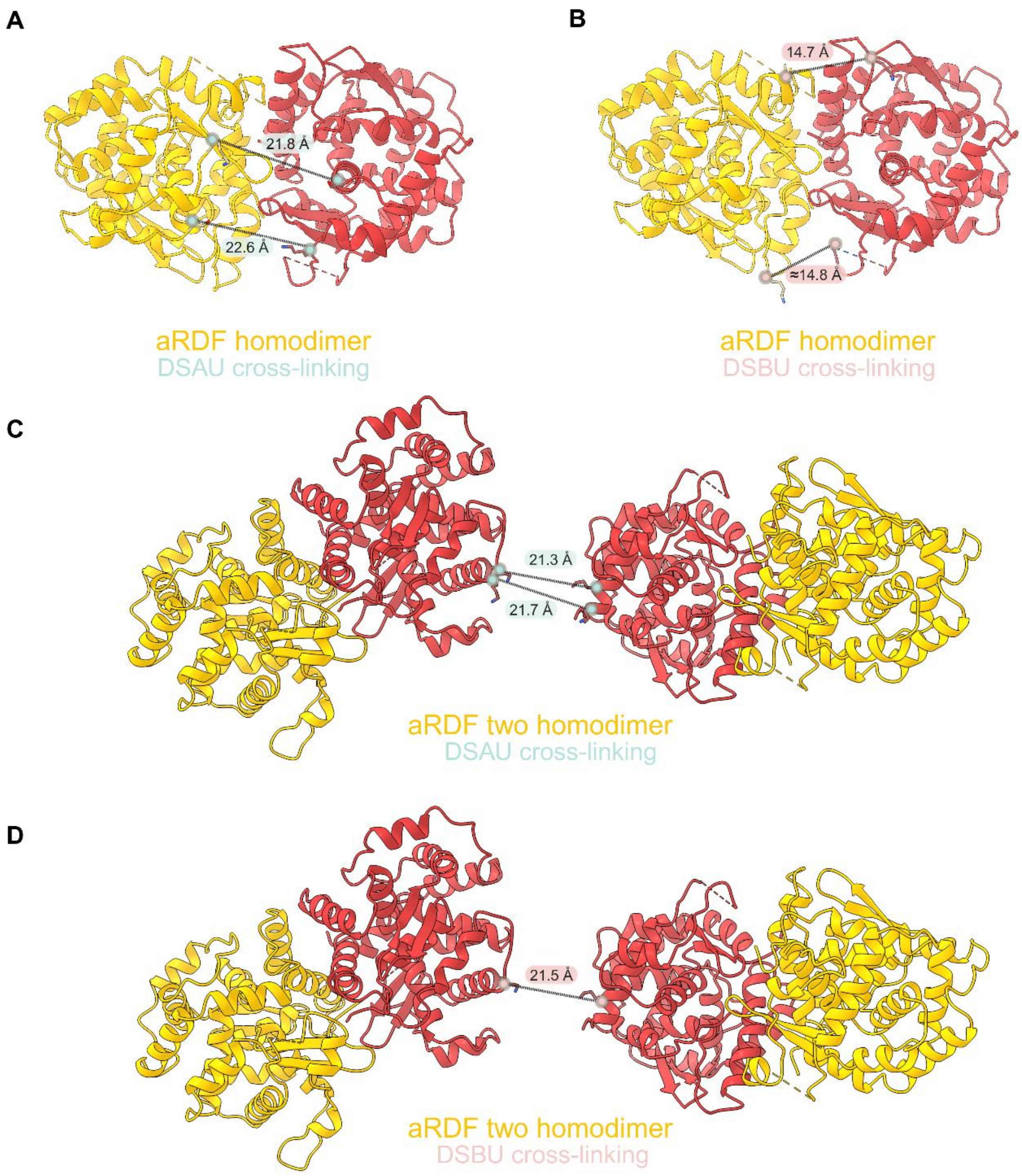

Supplementary Figure S5. Interprotein cross-linking within the homodimer of aRDF or between two homodimers of aRDF. (**A**) DSAU cross-linking within the homodimeric aRDF (cyan) and (**B**) DSBU cross-linking within the homodimeric aRDF (pink). (**C**) DSAU cross-linking within two homodimeric aRDF structures (cyan) and (**D**) DSBU cross-linking within two homodimeric aRDF structures (pink) in the 30S-aRDF complex. Selected cross-links are mapped onto the structure of aRDF, that are within the expected distance range of a cross-linker, with dotted lines indicating connections between residues and approximate distances in Å. The cross-linking experiments were conducted for aRDF alone (for panels A, B) and aRDF within the 30S-aRDF complex (for panels A-D); see also Supplementary Tables S3 and S4.

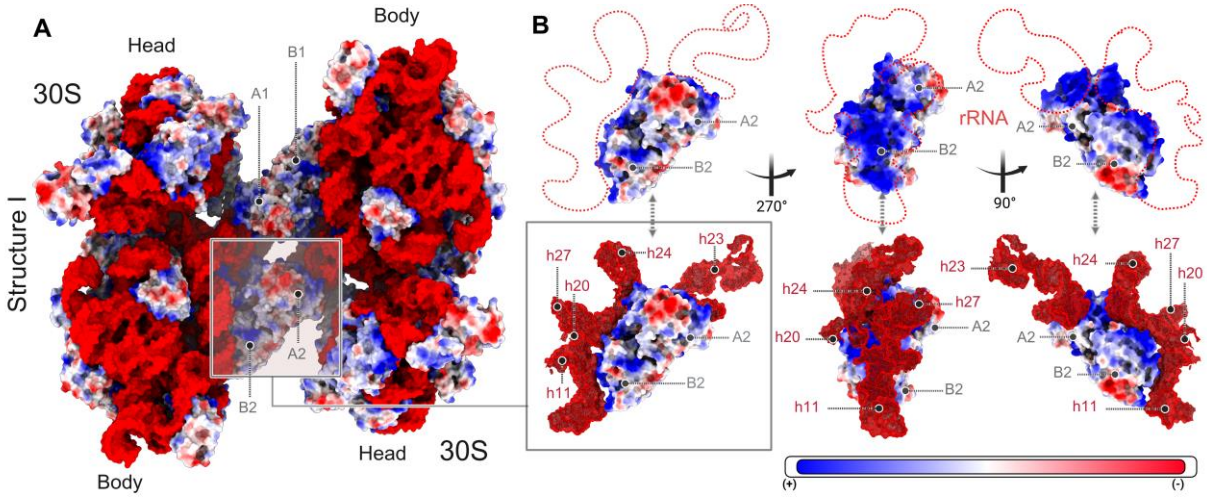

Supplementary Figure S6. Surface charge distribution of the interaction between 16S rRNA and aRDF protein. (**A**) Coulombic electrostatic potential (ESP) surface representations of the 30S-30S complex with two aRDF homodimers. Surfaces are colored according to electrostatic potential, ranging from red (negative potential), through white (neutral), to blue (positive potential); 10 to -10 kT/e^{-}. (**B**) Multiple views of the rRNA embedded on the surface of aRDF homodimer. Dotted lines indicate the respective positions of the rRNA on the aRDF homodimer. The rRNA helices involved in the interaction surface are labeled accordingly and highlighted in red mesh.

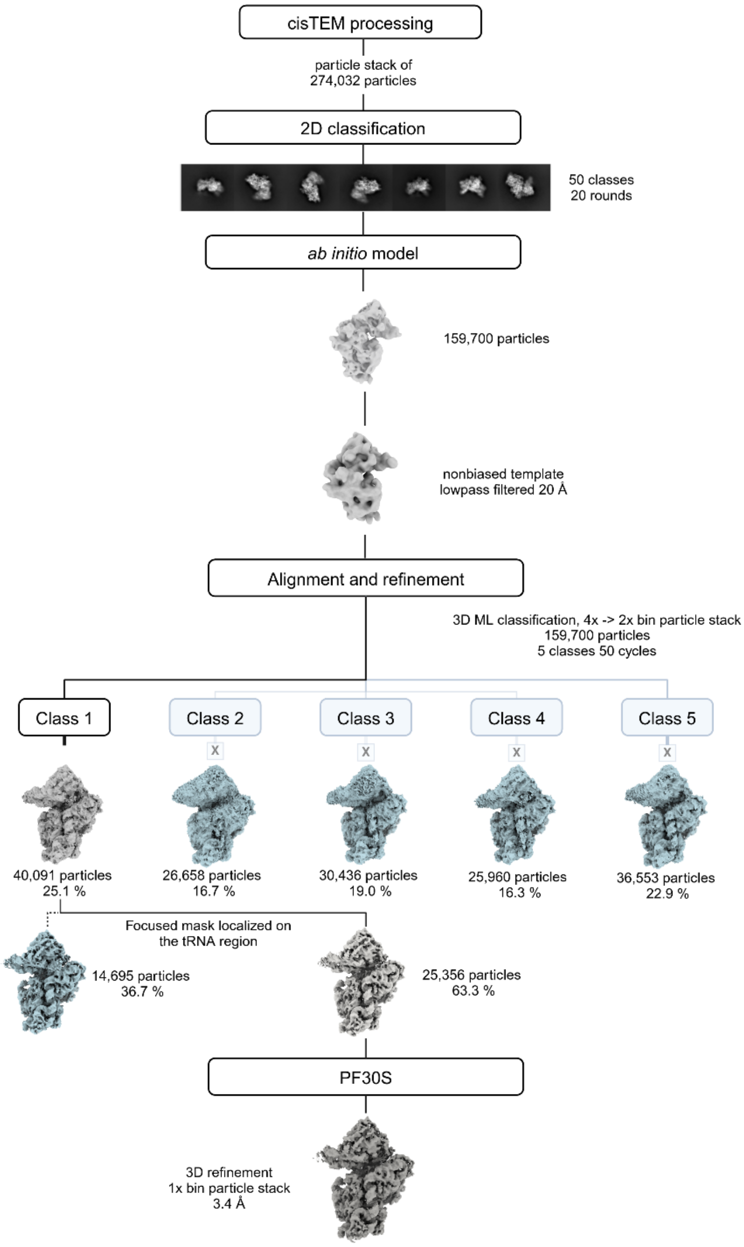

Supplementary Figure S7. Cryo-EM data classification scheme for *P. furiosus* 30S control (PF30S). The final map used in structural modeling is shown in grey (for 30S), while all other unused maps are depicted in blue.

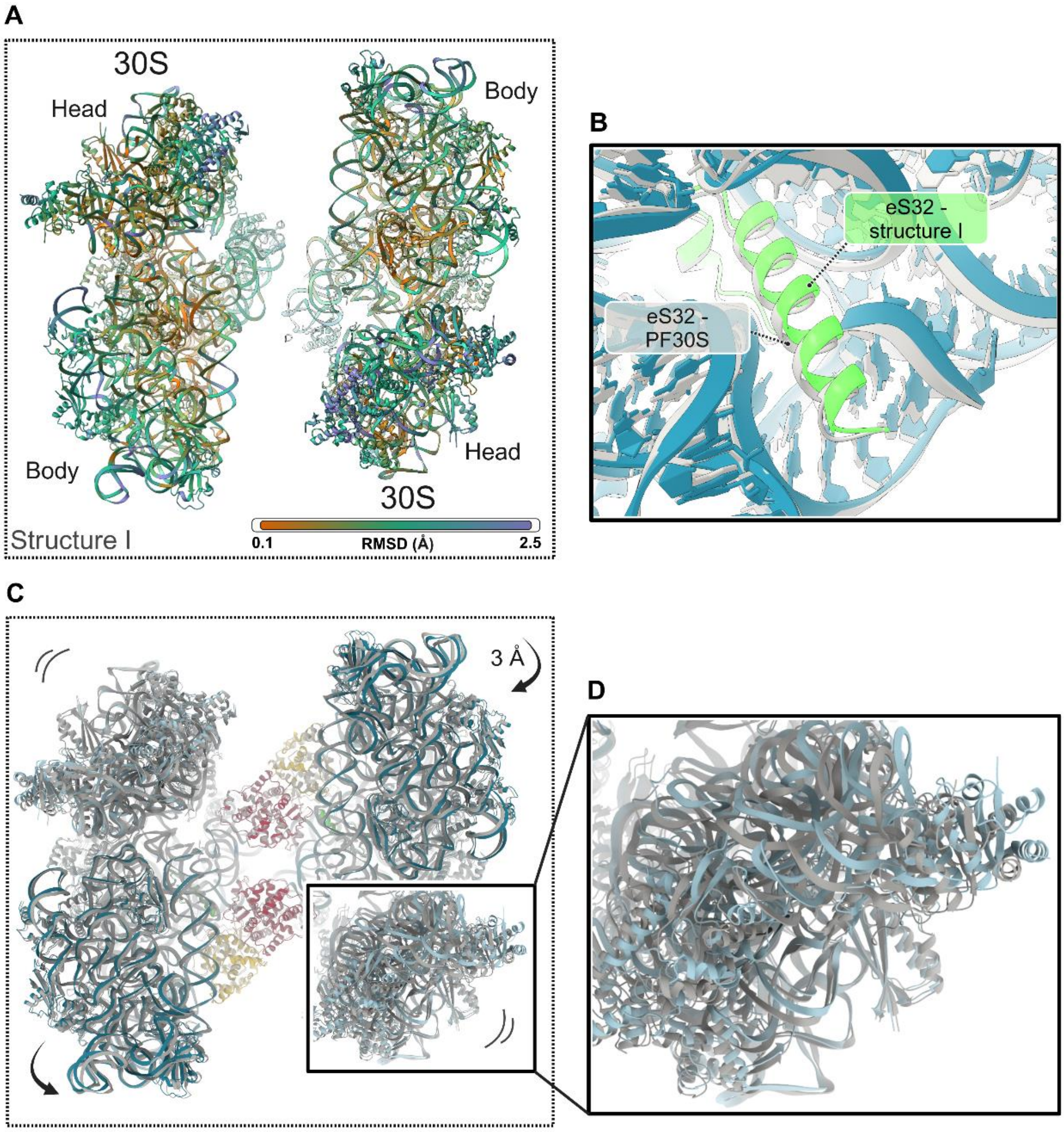

Supplementary Figure S8. Dynamic movement of the 30S-aRDF complex, along with alignment to PF30S control. (**A**) Structure I colored according to the RMSD deviation of the PF30S control. The PF30S control structure was aligned to Structure I using the PF30S body as the central point for alignment. (**B**) Close-up view of the eS32 protein in Structure I (lime) and PF30S control (grey), demonstrating the occupancy of the eS32 protein in the same region as in the PF30S control. (**C**) Structure I (in color code as in Fig. 2c) superimposed on Structure II (grey), revealing a PF30S body movement of 3 Å. Structure II was aligned to Structure I using two aRDF homodimers as the central point for alignment. (**D**) Close-up view of the PF30S head rotation by 4º.

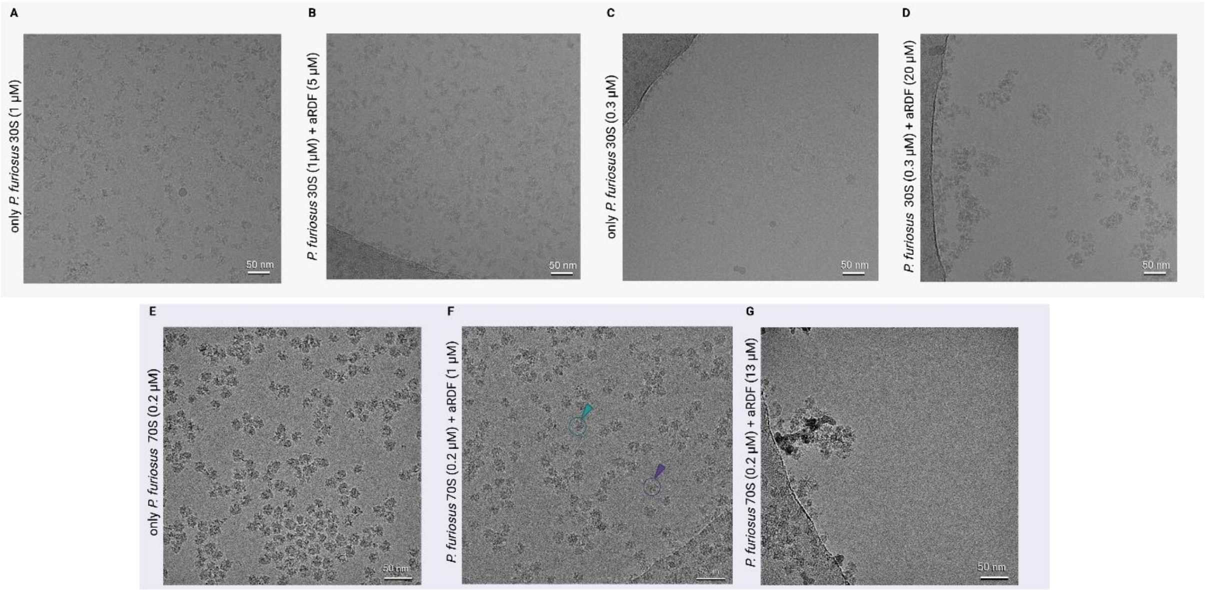

Supplementary Figure S9. 30S subunit and 70S samples with and without aRDF protein. (**A**) Cryo-EM micrograph showing a 1 µM 30S sample. (**B**) Cryo-EM micrograph illustrating a 1 µM 30S sample in the presence of 5 µM aRDF protein. (**C**) Cryo-EM micrograph showing a 0.3 µM 30S sample. (**D**) Cryo-EM micrograph illustrating a 0.3 µM 30S sample with an excess concentration of aRDF protein (approximately 20 µM). (**E**) Cryo-EM micrograph showing a 0.2 µM 70S sample. (**F**) Cryo-EM micrograph illustrating a 0.2 µM 70S sample in the presence of 1 µM aRDF protein. The 30S subunit particle is indicated by a cyan arrow, while the 50S subunit is marked with a violet arrow. (**G**) Cryo-EM micrograph illustrating a 0.2 µM 70S sample with an excess concentration of aRDF protein (approximately 13 µM).

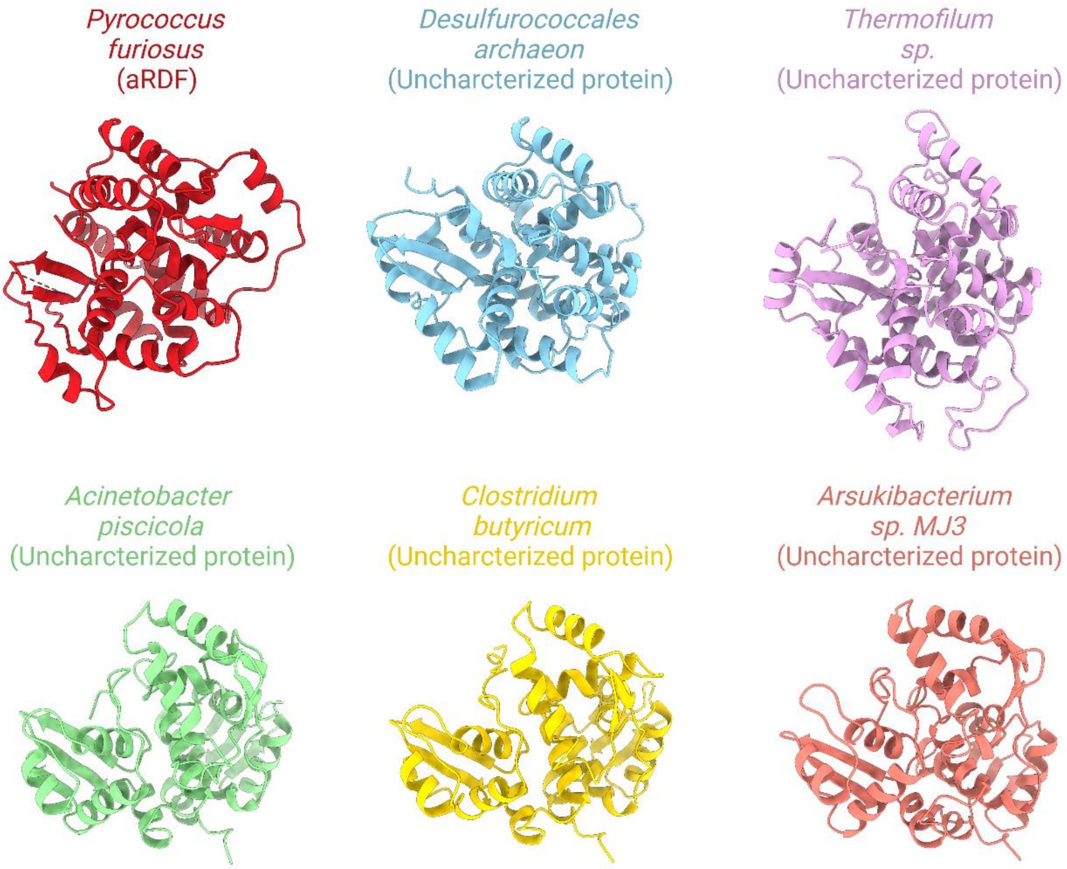

Supplementary Figure S10. aRDF-like proteins. Different uncharacterized proteins exhibiting similar fold to aRDF. The protein fold search was generated using Foldseek and Alphafold.
